# Inhibition of Hypersialylation in Human Intervertebral Disc Degeneration Modulates Inflammation and Metabolism

**DOI:** 10.1002/advs.202506669

**Published:** 2025-11-14

**Authors:** Kieran Joyce, Aert F. Scheper, Aung Myat Phyo, Roisin O’ Flaherty, Richard Drake, Aiden Devitt, Martina Marchetti‐Deschmann, Radka Saldova, Abhay Pandit

**Affiliations:** ^1^ CÚRAM Research Ireland Centre for Medical Devices Biomedical Sciences Building University of Galway Galway H91 W2TY Ireland; ^2^ School of Medicine University of Galway Galway H91 TK33 Ireland; ^3^ Department of Chemistry Maynooth University Maynooth W23 F2K8 Ireland; ^4^ Department of Cell and Molecular Pharmacology Medical University of South Carolina Charleston South Carolina 29425 USA; ^5^ Institute of Chemical Technologies and Analytics TU Wien Vienna 1060 Austria; ^6^ National Institute for Bioprocessing Research and Training (NIBRT) Dublin A94 X099 Ireland; ^7^ School of Medicine College of Health and Agricultural Science University College Dublin Dublin D04 C1P1 Ireland

**Keywords:** glycosylation, inflammation, intervertebral disc, sialylation

## Abstract

Intervertebral disc (IVD) degeneration is a major cause of low back pain (LBP), a significant global health burden. While glycosylation plays a key role in cellular signaling and inflammation, its role in IVD degeneration remains poorly understood. This study characterizes glycan alterations in human healthy and degenerated IVDs using glycomic (UPLC‐MS, MALDI‐IMS) and proteomic (LC‐MS) analyses, combined with functional studies. These results identify hypersialylation, especially α‐2,6‐linked sialic acid, as a prominent feature of degenerated IVDs. In vitro inhibition of sialylation (3Fax‐peracetyl Neu5Ac) in nucleus pulposus cells demonstrates reduced oxidative stress and inflammatory signaling, indicating a functional role for hypersialylation in IVD pathology. Targeting glycosylation pathways, notably sialylation, emerges as a promising therapeutic strategy for IVD degeneration.

## Graphical abstract

Glycosylation, specifically hypersialylation, is identified as a critical factor in human intervertebral disc (IVD) degeneration—a major cause of low back pain. This study demonstrates that inhibiting sialylation reduces inflammation and oxidative stress in IVD tissues, suggesting new therapeutic possibilities. These findings highlight glycan modifications as promising targets for improving outcomes in degenerative disc diseases.

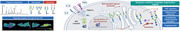

## 1 Introduction

Low back pain, a global health concern, imposes a significant socio‐economic burden on society. A key contributor to this pain is intervertebral disc degeneration (IVDD), a complex condition characterized by intricate alterations in the extracellular matrix, cell phenotype, and inflammatory responses. While tissue engineering solutions have been developed to address the underlying mechanisms of IVDD and restore disc physiology, the role of glycosylation in this pathogenesis remains largely unexplored. This study investigates potential targets and mechanisms for augmentation to explore the role of glyco‐modulatory molecules for biomaterial systems for intervertebral disc (IVD) regeneration.

Glycosylation is a post‐translational modification involving the synthesis of glycans, addition and modification of these oligosaccharides to lipids, proteins, and RNA by glycosyltransferases and glycosylhydrolases/glycosidases, generating a diverse range of glycoconjugates.^[^ ^1^ ^]^ Glycosylation patterns provide insight into the temporal and spatial regulation of glycans in tissue inflammation and degeneration.^[^ ^1^ ^]^ These glycans are pivotal in protein folding, trafficking, receptor expression, activation, intracellular signaling, and immunomodulation.^[^ ^2^ ^]^

Previous research has laid the foundation by investigating glycan expression at the histological level in embryological notochord and fetal intervertebral discs.^[^ ^3^, ^4^, ^5^ ^]^ However, advancements in glycoprotein extraction and analysis have allowed for a more precise exploration of glycosylation in connective tissues, such as cartilage.^[^ ^6^, ^7^, ^8^ ^]^ The glycosylation profile of IVD has been studied in murine,^[^ ^9^ ^]^ bovine^[^ ^10^ ^]^ and ovine^[^ ^11^ ^]^ models for injury and degeneration. These studies have briefly investigated the overall glycosylation motif expression using lectin arrays. Nevertheless, a significant gap exists in the field regarding the comprehensive analysis of the human glycome, encompassing *N*‐glycans and other glycan species. Given the central role of glycans in metabolism, proliferation, and differentiation mechanisms, they may play a critical role in regenerative responses within the IVD, differentiating healthy discs from aged, degenerated ones.

To further unravel the role of glycans in degeneration, we turned our attention to sialylation, a type of glycosylation motif that plays a key role in cell‐to‐cell interactions, cell signaling, and inflammation.^[^ ^12^, ^13^ ^]^ Previous studies have established the co‐localization of sialic acid moieties at the level of chondrocytes in articular cartilage^[^ ^8^, ^14^, ^15^ ^]^ and thereby recognized them as key components of the interactions of these cells with the extracellular matrix (ECM). Moreover, the role of dysregulation of sialic acids and sialic acid linkage on the cell surface, which is fundamental for cell‐to‐matrix interactions and mechanosensing, was characterized.^[^ ^15^ ^]^

**Figure 1:**
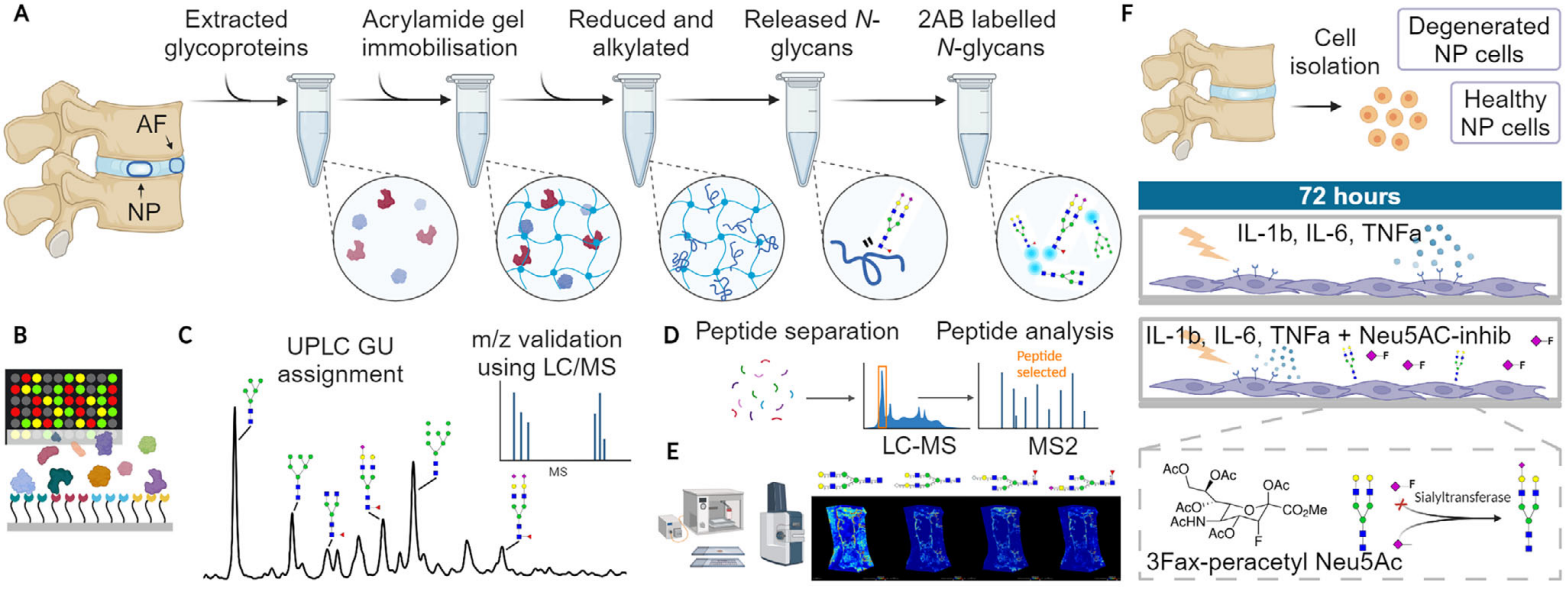
Schematic representation of the experimental design and procedures. A) Glycoprotein isolation and enzymatic *N*‐glycan release. B) lectin microarray of intact glycoproteins. C) Quality assessment, assignment and quantification of *N*‐glycans. D) Proteomic analysis workflow highlighting upregulated inflammatory pathways in the degenerated IVD. E) MALDI‐TOF‐MSI: glycan ion maps characterize the spatial presentation of *N*‐glycans. F) Workflow for in vitro model of IVD degeneration and Neu5Ac‐inhib studies.

In this study, we set out two primary objectives: 1) to comprehensively characterize the *N*‐glycome of the human disc with spatial and temporal resolution to investigate the dysregulation of glycans in degeneration. We sought to understand these changes in the context of the associated proteome and the overall glycome (including *O*‐glycans, glycosphingolipids, etc.), combining dedicated *N*‐glycan analysis and lectin microarray. Subsequently, upon confirming the presence of hypersialylation, we aimed to uncover the functional role of sialylation in degeneration through a mechanistic study using a small molecule inhibitor of sialylation (3Fax‐peracetyl Neu5Ac; Neu5Ac‐inhib). Our collective findings suggest that the glycosylation response, especially sialylation, plays a functional role in degeneration.^[^ ^12^ ^]^ Furthermore, sialylation inhibition can potentially reduce inflammation and oxidative stress, making it a promising adjunct to regenerative therapies, paving the way for the first generation of glyco‐functionalized therapies for IVD regeneration. The experimental design for this study is outlined in **Figure** **1**.

## 2 Results

### 2.1 *N*‐Glycome of Human IVD

**Figure 2:**
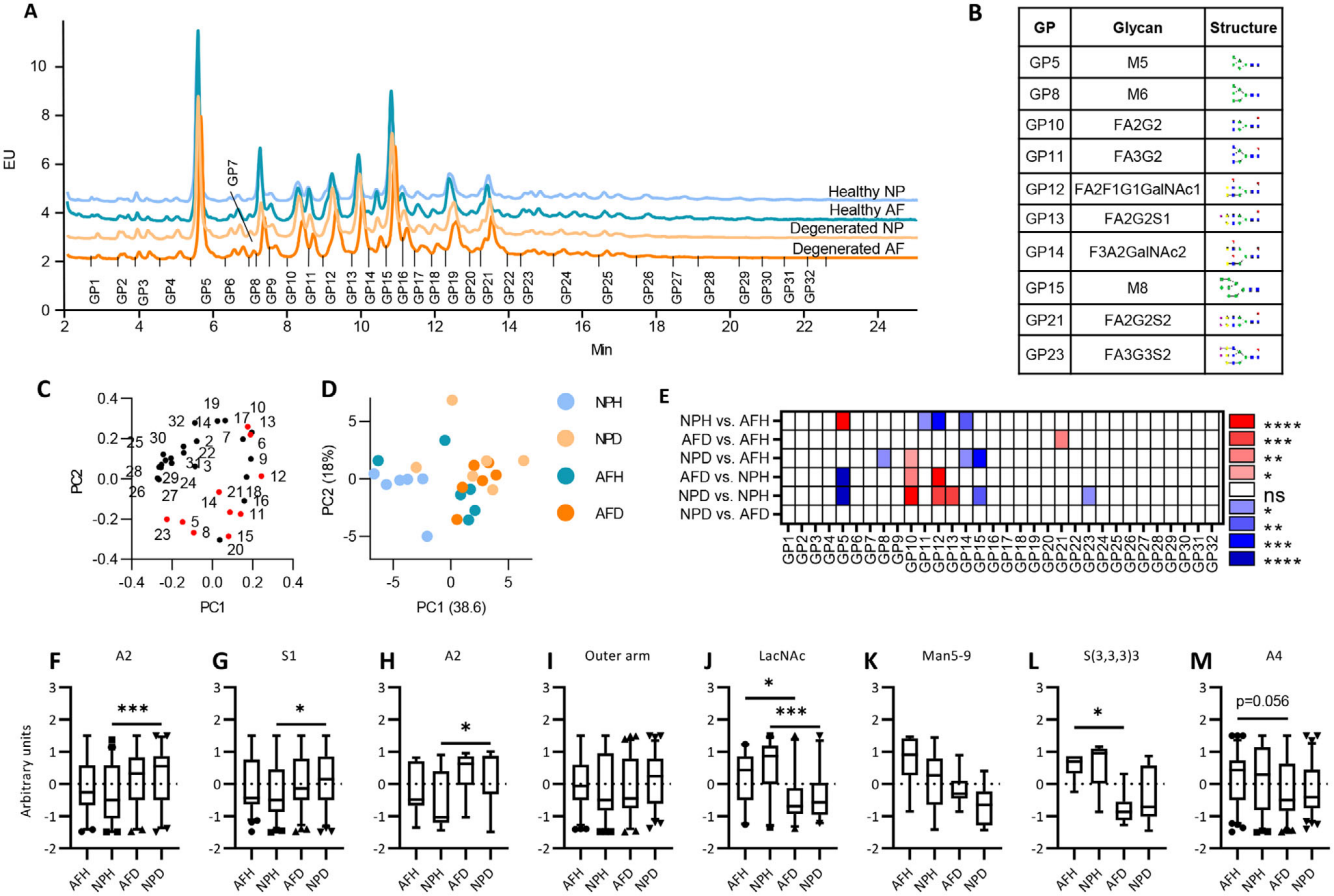
Biantennary and oligomannose glycans are differentially regulated in IVD degeneration, demonstrated using HILIC‐UPLC. A) HILIC‐UPLC chromatograms for *N*‐glycans isolated from human IVD samples (*n*=6 for each group). 32 glycan peaks (GP) identified across all samples. Detailed composition of each peak is described in Table S1 (Supporting Information). B+E) Summary of significantly altered peaks across group comparisons B) and overview of the most abundant glycans in each significantly altered peak. Red indicates a significant increase and blue indicates a significant decrease in the relative area under the curve for each GP,. (E). C+D) Principal component analysis and GP contributions to loadings. Red indicates significantly different GPs. F‐M) Individual glycans were grouped into the following glycosylation traits; biantennary +/‐ outer arm fucosylation (A2+/‐F) (F), monosialylated glycans (S1)(G), biantennary excluding outer arm fucosylated species (A2)(H), outer arm fucosylated (outer arm)(I), lactosaminylated (LavNAc)(J), higher oligomannose‐from Man5 to Man9 (Man5‐9)(K), trisialylated, alpha 2,3‐linked (S(3,3,3)3)(L), tetraantennary (A4)(M) (Median, IQR, min‐max). Two‐way ANOVA, Tukey's post hoc test, *p <0.05, **p <0.01, ***p <0.001, ****p <0.0001.

To unveil key glycans dysregulated in degeneration, the *N*‐glycome from pooled healthy (Pfirrmann Grade I, *n* = 6) and degenerated (Pfirrmann Grade IV/V, *n* = 6) IVD samples was released using the previously described method.^[^ ^16^ ^]^ Six biological replicates from healthy and degenerated annulus fibrosus (AF) and nucleus pulposus (NP) were run undigested, where biological covariance varied from 5 to 70% (average<20%) (Dataset S2, Supporting Information). 32 peaks were identified as common across all individual sample replicates (**Figure** **2**A). The peaks with over 20% covariance accounted for less than 5% of *N*‐glycans, occurring at the beginning or end of the profile with a very low‐intensity signal, where the small variability accounts for a greater proportion of peak variability, increasing the covariance. Technical replicates demonstrated <5% covariance to ensure reproducibility as previously described.^[^ ^16^, ^17^ ^]^

Two hundred eighty three unique glycan species were identified, with 270 species common across all *N*‐glycan populations (Dataset S2, Supporting Information). These were assigned to 48 glycan peaks common to the pooled samples with a summary of all assigned glycans and relative percentage abundance based on exoglycosidase digestion profiles and confirmed by LC‐MS (GP1 – GP48; see Dataset S2, Supporting Information). Significant differences in peak area were observed in GP5, 8, 10‐15, 21, and 23 (Figure 2B,E). Glycan isoforms M5 (GP5), M8 (GP15), and FA3G3S2 (GP23) were significantly decreased in degenerated NP (NPD) vs healthy NP (NPH), while FA2G2 (GP10), FA2F1G1GalNAc1 (GP12), and FA2G2S1 (GP13) were significantly increased. FA2G2S2 (GP21) was the only peak that significantly decreased in healthy AF (AFH) vs NPH. M5 (GP5) was increased in AFH vs NPH, while FA3G2 (GP11), FA2F1G1GalNAc1 (GP12), and FA2F2GalNAc2 (GP14) were all decreased. No significant differences were observed between NPD and degenerated (AFD), suggesting that the glyco‐signatures of NP and AF converge in degeneration, as observed in the lectin microarray analysis (Figure S1, Supporting Information).

Principal component analysis (PCA) of glycosylation motifs (Figure S3A,B, Supporting Information) and all 283 identified glycans (Figure S3C–E, Supporting Information) revealed distinct clustering of healthy and degenerated tissues. PC1 provides a strong separation of healthy tissue, where oligomannose, tetraantennary, lactosamine, and non‐sialylated glycans are strong contributors toward a healthy phenotype, while biantennary, outer arm fucose, sialylated, and complex glycans contribute to the degenerated phenotype. Overall, monosialylated (S1) and biantennary (A2) glycans increased significantly in degenerated tissues vs healthy IVD (Figure 2F–H). Outer arm fucosylation also trended to increase in degenerated NP vs healthy controls (Figure 2I). Conversely, oligomannose (Man5‐9), tetraantennary (A4), and lactosamine‐terminated (LacNAc) glycans decreased in degenerated tissues, indicating a shift from branched high‐molecular‐weight glycans to small monoantennary and biantennary glycan species (Figure 4J,K,M). Sialylation linkages were characterized by differentiating between NAN1 and ABS exoglycosidase digestions to discriminate α‐^[^ ^2^, ^3^ ^]^ vs α‐^[^ ^2^, ^6^ ^]^ linkages. α‐^[^ ^2^, ^6^ ^]^ sialylation increased in degenerated IVD tissues from 19.57% and 19.46% in healthy AF and NP to 24.05% and 23.10% in degenerated AF and NP, respectively (Dataset S2, Supporting Information).

### 2.2 Spatial Glycan Analysis

**Figure 3:**
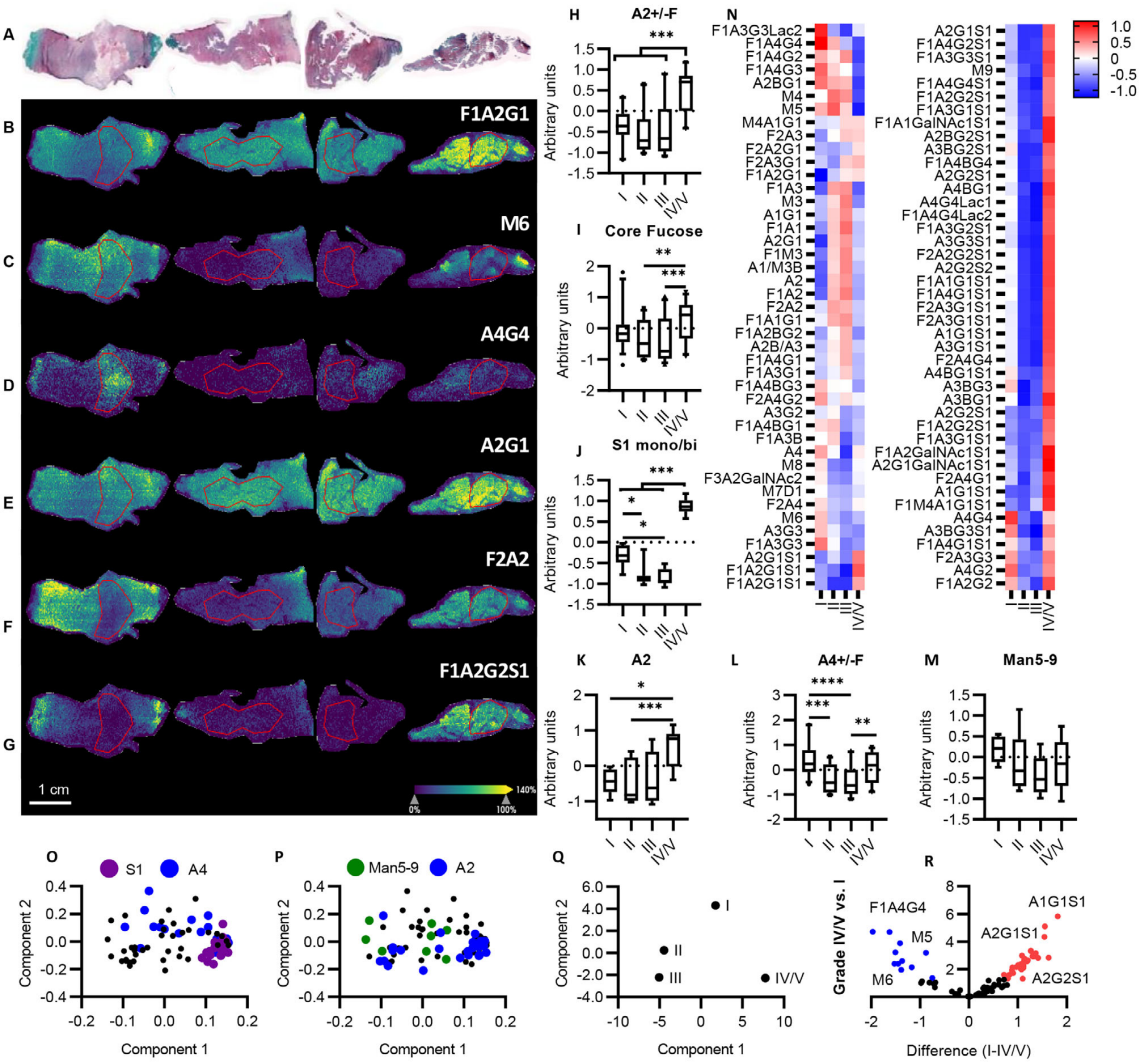
*N*‐glycan imaging of the human IVD in degeneration. A) Haematoxylin, Safrinin‐O, Fast Green FCF histological assessment, n=6 B‐G) Glycan ion maps of representative glycan traits. Region of interest, nucleus pulposus, marked by red outline. H‐M) Individual glycans were grouped into the following glycosylation traits; biantennary +/‐ outer arm fucosylation (A2+/‐F) (H), core fucosylated glycans (I), mono/biantennary sialylated glycans (S1 mono/bi)(J), biantennary excluding outer arm fucosylated species (A2)(K), tetraantennary +/‐ outer arm fucosylation (A4+/‐F) (L), higher oligomannose‐from Man5 to Man9 (Man5‐9)(M). N) Z‐score normalized heatmap of glycan abundance across grades. O‐Q) Principal component analysis separating grades of IVD degeneration and loadings highlighting the contribution of O) sialylated and tetraantennary glycans, P) Man5‐9 and biantennary glycans toward each condition. (Median, IQR, min‐max). R) Volcano plot separating expressed glycans in grades I and IV/V. Two‐way ANOVA, Tukey's post hoc test, *p <0.05, **p <0.01, ***p <0.001 ****p <0.0001.

Matrix‐assisted laser desorption inoisation‐imaging mass spectrometry (MALDI‐IMS) of *N*‐glycans was used to determine the spatial regulation of *N*‐glycans in the human IVD, as described by Rebelo et al.^[^ ^18^ ^]^ Overall, 77 *N*‐glycans were detected on MALDI MSI (Dataset S3, Supporting Information). Distinct differences in glycan patterning were observed between grades of degeneration (**Figure** **3**). Spatial characterization of *N*‐glycans highlights NP‐specific tetraantennary glycans and AF NP‐specific biantennary glycans in healthy IVD (Figure 3d,F). This spatial distinction was lost in degeneration, correlating with histological loss of AF/NP differentiation. Oligomannose and tetraantennary *N*‐glycan structures were more abundant in Grade I and II, while mono/biantennary, with or without sialylation, were more abundant in Grade IV/V (Figure 3H–M). PCA demonstrates separation of degenerated IVD glycome in which biantennary sialylated glycans contribute to degenerated IVD component separation and oligomannose and tetraantennary glycans contribute to a healthy glycome (Figure 3O,P).

### 2.3 RNA Sequencing Revealed Inflammatory Pathway Mediation in Sialylation Inhibited Nucleus Pulposus Cells

To characterize the cellular response to sialylation inhibition in IVD degeneration, an in vitro model using human nucleus pulposus cells was established as previously described.^[^ ^12^ ^]^ Healthy cells (H‐CON) were stimulated with inflammatory cytokines (IL‐1β, TNF‐α, and IL‐6) to establish a pro‐inflammatory response (H‐CYTKN). The treatment group (H‐TREAT) included the co‐incubation of 3Fax‐peracetyl Neu5Ac (Neu5Ac‐inhib) in the pro‐inflammatory culture conditions. In parallel, degenerated cells (D‐CON) were also treated with Neu5Ac‐inhib (D‐TREAT), where no cytokines were used in this culture, as it is presumed that these cells are conditioned to a chronic pro‐inflammatory state.

**Figure 4:**
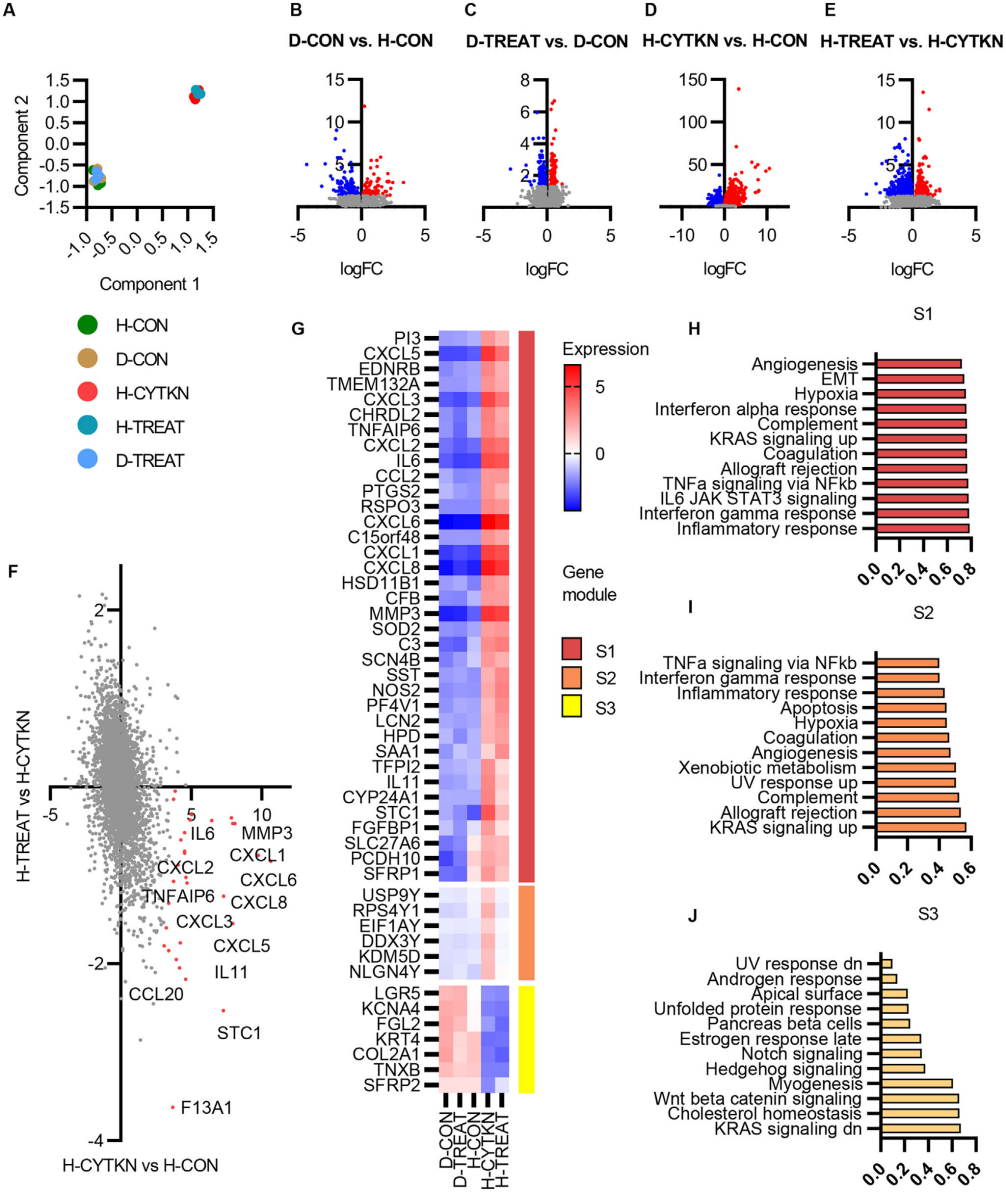
Transcriptional data from human NP cells under inflammatory and glycosylation inhibitor‐treated conditions. A) PCA analysis of five conditions: H‐CON (healthy NP cells), D‐CON (degenerated NP cells), H‐CYTKN (healthy NP cells +cytokine stimulation), H‐TREAT (healthy NP cells +cytokine stimulation + 3F‐peracetyl Neu5Ac), and D‐TREAT (degenerated NP cells + 3F‐peracetyl Neu5Ac). *n*=3 B–E) differential gene expression across experimental conditions; Upregulated genes are highlighted in red, and downregulated genes are highlighted in blue. False Discovery Rate <0.05. F,G) Differential expression analysis comparing common genes dysregulated under cytokine stimulation and Neu5Ac‐inhib. H,I) Grouped subset analysis with associated pathway analysis using unsupervised hierarchal clustering (correlation (R)).

An overview of the sample groups is provided through a principal component analysis (PCA), where components separate groups based on cytokine stimulation only. Few DEGs were identified when comparing degenerated and healthy NP cells (D‐CON vs H‐CON; up 646, down – 715) (**Figure** **4**B), while 1575 DEGs were confirmed in degenerated cells treated with Neu5Ac‐inhib (D‐TREAT vs D‐CON); up – 712, down – 863) (Figure 4C) (Dataset S5, Supporting Information). A total of 5174 differentially expressed genes (DEGs) were identified in healthy NP cells under cytokine stimulation (H‐CYTKN vs H‐CON; up – 2709, down‐2465) (Figure 4D). Similarly, 2881 DEGs were identified when inflamed cells were treated with Neu5Ac‐inhib (H‐TREAT vs H‐CYTKN; up – 1151, down – 1730) (Figure 4E). Pro‐inflammatory genes upregulated in cytokine‐stimulation were downregulated by Neu5Ac‐inhib (Figure 4F,G).

**Figure 5:**
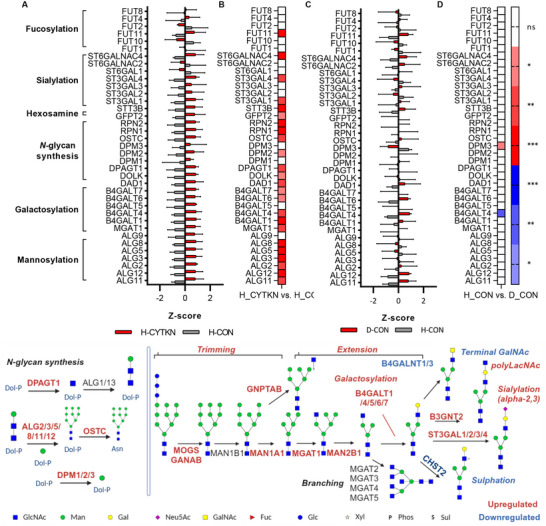
Overview of glycosylhydrolases and glycosyltransferases in *N*‐glycan synthesis and decoration under cytokine stimulation in human NP cells. A+B) Glycosyltransferase expression in cytokine stimulation, significance interval presented in the heatmap, n=3, *p <0.05, **p <0.01, ***p <0.001 (B). C+D) Glycosyltransferase expression in degeneration, significance interval presented in heatmap, Mean ± SEM, **p <0.01, ***p <0.001 (D). Upregulated (red) and downregulated (blue) genes are highlighted, two‐sided t‐test, FDR <0.05.

Pathways such as Inflammatory response, IL6 JAK STAT, TNF‐α, and complement activation are enriched in upregulated subunit clusters in cytokine stimulation (Figure 4I). There was a significant decrease in TNF‐α signaling, inflammatory response, and epithelial‐mesenchymal transition in Neu5Ac‐inhib treated cells (Figure S4A, Supporting Information). The same response to sialylation inhibition is not observed in non‐stimulated degenerated cells. There is an upregulation of glycosyltransferases in cytokine‐stimulated cells vs healthy NP cells (**Figure** **5A**), however, this upregulation is not observed in degenerated NP cells (Figure 5B). This is likely due to the resolution of cellular stress in culture conditions vs in vivo conditions.

## 3 Discussion

Extensive cellular glycosylation analyses of the human intervertebral disc have been limited in previous research efforts. However, it is increasingly recognized that glycosylation plays a vital role in various biological processes. Developmental morphological changes are often correlated with alterations in carbohydrate expression on cell surfaces and in the extracellular matrix (ECM). Studies in both rat and human models have suggested that disruptions in normal glycosylation patterns may contribute to developmental malformations.^[^ ^8^, ^19^, ^20^ ^]^ Despite this, a notable gap exists in understanding glycosylation patterns during human vertebral morphogenesis, maturity, and disease.^[^ ^21^ ^]^

Sialylation, specifically α‐^[^ ^2^, ^6^ ^]^ sialylation through ST6GAL1 is required for somatic cell reprogramming, and its downregulation is associated with decreased reprogramming efficiency.^[^ ^22^ ^]^ Therefore, the observed upregulation of sialylation and fucosylation within the IVD may signify phenotypic changes in resident cells. Similarly, the upregulation of outer arm fucosylation has been indicated in the functional regulation of TIMPs. While TIMP1 expression increases in the degenerated disc, as shown here and previously described,^[^ ^23^ ^]^ it is plausible that its MMP‐binding capacity is compromised due to increased outer arm fucosylation, altering matrix catabolism. It is crucial to note that the *N*‐glycosylation site function is unique to each glycoprotein sequence. This uniqueness, coupled with regulating the Golgi *N*‐glycan‐branching pathway, determines surface glycoprotein levels.^[^ ^24^ ^]^ Notably, this pathway is highly sensitive to intracellular hexosamine flux, particularly in the production of tri‐ and tetraantennary *N*‐glycans. These complex glycans have been shown to bind to galectins and form a molecular lattice that hinders glycoprotein endocytosis, thereby regulating surface protein expression.^[^ ^24^ ^]^

In recent years, there has been a growing interest in developing glycosylation inhibitors as potential therapeutic agents.^[^ ^25^ ^]^ In this study, we sought to comprehensively characterize the *N*‐glycome of the human intervertebral disc (IVD) with spatial and temporal resolution. Our primary goal was to investigate the dysregulation of glycans in the context of IVD degeneration. To achieve this, we employed a multifaceted approach that combined lectin histochemical analysis, glycan profiling, and proteomic analysis.

The results of the initial lectin histochemical analysis in this study were also consistent with previous glycosylation profiling in bovine^[^ ^26^ ^]^ and murine^[^ ^27^ ^]^ IVD injury models and ovine IVD ageing.^[^ ^11^ ^]^ Increased expression of sialylation and fucosylation on ultra performance liquid chromatography‐mass spectrometry (UPLC‐MS) aligns with the observations made with lectin histochemistry (SNA; α‐^[^ ^2^, ^6^ ^]^‐sialylation, UEA; α‐^[^ ^1^, ^2^ ^]^‐linked fucose) (Figure S1G–M, Supporting Information) reflecting the increased expression of sialyltransferases/fucosyltransferases (ST3GAL1/4, ST6GALNAC4, FUT11) in cytokine‐induced inflammation, supported by previous studies in inflammation in fibrous tissues.^[^ ^14^, ^26^ ^]^ The upregulation of sialyltransferases (ST6GAL1) and expression of α‐^[^ ^2^, ^3^ ^]^ and α‐^[^ ^2^, ^6^ ^]^‐sialylated glycosylation motifs have been characterized in human cartilage degeneration, as indicated by the binding of several lectins.^[^ ^27^ ^]^ Proteomic analysis revealed an upregulation in apoptosis, p53 pathway, and complement (Figure S2G–J, Supporting Information), which has been characterized previously to upregulate the expression of sialyltransferases and fucosyltransferases in connective tissues.^[^ ^28^ ^]^ The observed alterations in ECM constituents and glycoproteins may contribute to the glycosylation changes identified in the study.

**Figure 6:**
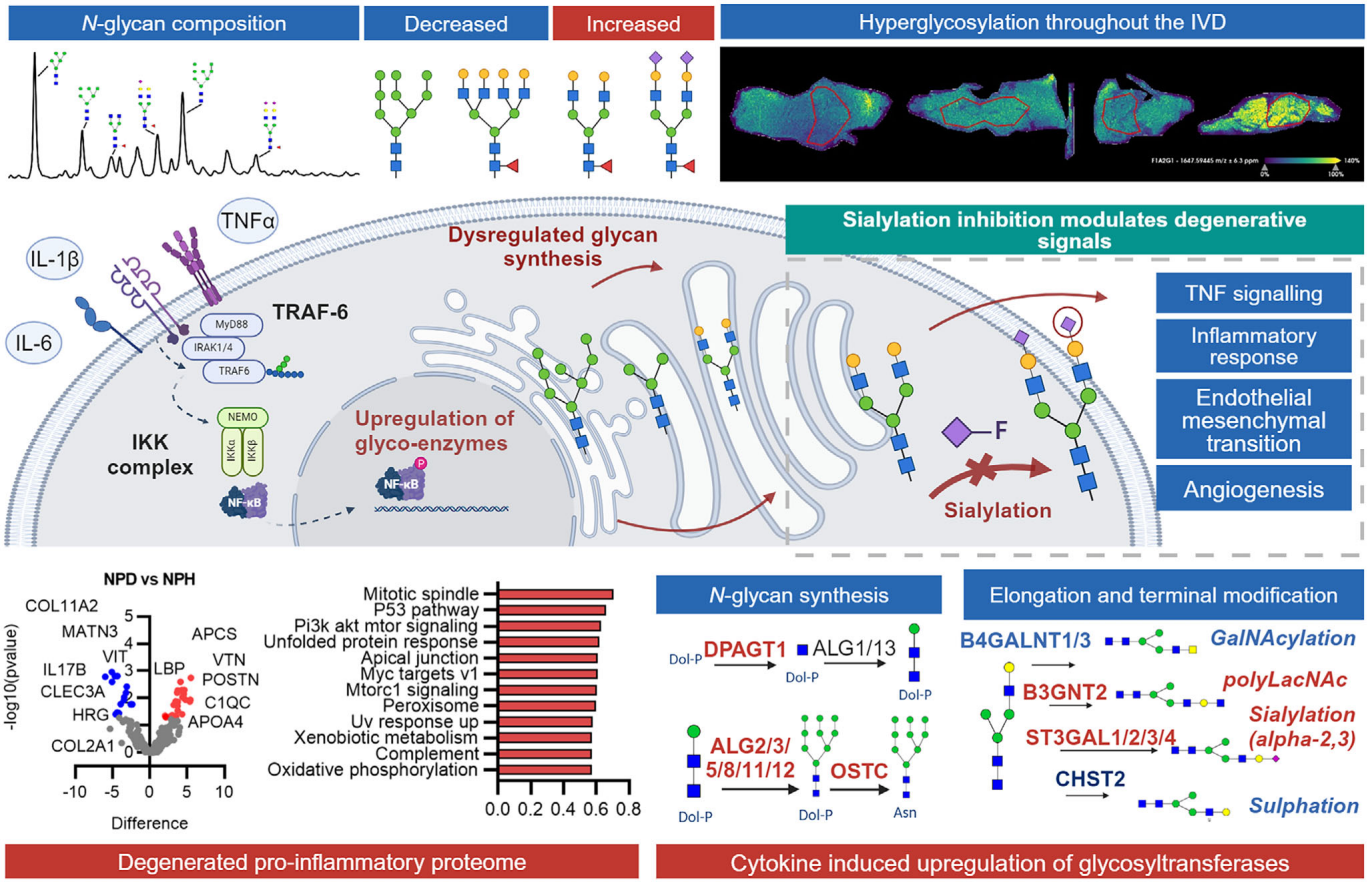
Glycosylation in IVD degeneration; cause and effect of aberrant glycan synthesis. Overview of glycomic and proteomic analysis in the context of IVD pathophysiology. The changes in glycosylation toward mono/biantennary sialylated glycans are associated with a pro‐inflammatory proteome. Inhibitor studies have demonstrated an inhibition in TNF and inflammatory response pathways in a hypo‐sialylated state.

In vitro, we explored how Neu5Ac‐inhib affects glycosylation inhibition through different gene set enrichment analyses of transcriptomic data. RNA sequencing analysis in the given experimental groups revealed activation of inflammatory signaling in cytokine‐stimulated NP cells. Co‐incubation of NP cells with Neu5Ac‐inhib altered the expression of signaling pathways associated with ECM organization, cell‐cell interactions, adherens junction, *N*‐glycan synthesis and processing, and various Gene Ontology (GO) term‐enriched pathways identified through pathway analysis. The resultant hyperglycosylation states identified in this model are likely due to the upregulation in glycan synthesis and turnover, yet specific sialyltransferases were also significantly upregulated. ST3GAL1, found to be upregulated with cytokine stimulation, has previously been shown to be upregulated in an in vitro model of osteoarthritis.^[^ ^29^ ^]^ Degenerated NP cells in vitro likely experience partial resolution of chronic stress due to culture conditions, masking some differences. In addition, degenerated NP cells are less susceptible to therapeutic responses, which is well established in the field and one of the greatest challenges to IVD regeneration. However, our MALDI‐IMS and glycomic analyses of native human IVD tissue clearly demonstrated hypersialylation in vivo (**Figure** **6**). Thus, while transcriptomic shifts are modest in degenerated NP cells in culture, the addition of glyco‐modulatory elements through delivery systems (in the form of cells or otherwise) would augment the host response.

Hallmark pathways such as TNF signaling, angiogenesis, IL6 JAK STAT3 signaling, and interferon‐gamma response are enriched in cytokine‐stimulated NP cells. The observed changes in gene expression patterns, particularly the downregulation of proinflammatory genes in response to Neu5Ac‐inhib, indicate the potential of glycosylation modulation to mitigate inflammation, a significant factor in IVDD pathogenesis. The mechanism for the observed reduction in inflammatory signaling was not elucidated in this study; however, a possible mechanism for this is the suppression of a proteomic response and synthesis of inflammatory signaling molecules through sialylation inhibition, thus reducing downstream signaling and paracellular signal propagation.

Studies have shown that nutrient flow, driven by highly active growth‐promoting receptors, can stimulate cell arrest and differentiation pathways. This effect is mediated by an increased presentation of sparsely decorated *N*‐glycoproteins with mono‐ and biantennary glycosylation.^[^ ^30^ ^]^ Notably, we observed a similar phenomenon in the human intervertebral disc (IVD), where a decrease in branching was evident. This suggests a close metabolic link between cellular growth regulation and the number and branching of *N*‐glycans. The degree of *N*‐glycan branching appears to serve as a molecular mechanism responsive to hexosamine flux, thereby modulating the metabolic transition between cellular growth and arrest. Importantly, this regulation seems to be influenced more by hexosamine flux and nutrient availability, particularly considering the reduced nutrient supply in degenerated IVDs, rather than being solely mediated by glycosyltransferase activity. Furthermore, the prevalence of oligomannose‐type structures in healthy tissue may indicate cellular pluripotency, as such structures are recognized as markers of pluripotent stem cells.^[^ ^31^, ^32^ ^]^ It is plausible that the greater abundance of oligomannose motifs in healthy tissue results from endoplasmic reticulum (ER) expansion during translational processes, rather than being indicative of ER stress.^[^ ^33^ ^]^

## 4 Conclusion

In conclusion, our comprehensive investigation into glycosylation patterns in intervertebral disc degeneration sheds light on the critical role of glycosylation in this complex process. We observed distinct alterations in glycan expression, notably hypersialylation and decreased branching, which are associated with IVD degeneration. These changes indicate phenotypic shifts in resident IVD cells and have implications for regulating inflammatory responses and extracellular matrix dynamics. Furthermore, the underlying altered proteomic expression provides several indications toward the interplay between proteomic and glycomic homeostasis in IVD degeneration. Overall, transcriptomic analysis of cytokine stimulation in NP cells elucidates the functional role of sialylation in inflammatory signaling. Our findings highlight the importance of glycosylation in IVD biology and suggest that targeting specific glycosylation motifs, such as sialylation, may hold therapeutic potential in mitigating inflammation associated with IVD degeneration. This approach to glyco‐modulation using a small‐molecule inhibitor demonstrates proof‐of‐concept of glyco‐modulation that can be incorporated into biomaterial systems. Overall, this research advances our knowledge of the glycomic mechanisms underlying IVD degeneration and opens new avenues for developing glyco‐functionalized therapies to restore disc physiology.

## 5 Experimental Section

### Collection and Processing of Human Tissues

Human tissues were collected from three local hospitals. These tissues were used for UPLC *N*‐glycan analysis, lectin microarray analysis, and proteomic analysis. Healthy human NP cells for transcriptomic analysis were isolated from discs procured during spinal realignment surgery for adolescent idiopathic scoliosis (donor age range: 9‐16 years old, Pfirrmann grade I, Male:Female 50:50). Degenerated cells were isolated from discs during discectomy and fusion surgery (donor age range: 36‐72 years old, Pfirrmann grade IV/V Male:Female 50:50). Degenerated intervertebral discs were harvested from patients with diagnosed intervertebral disc degeneration. These discs were procured during discectomy and microdiscectomy procedures. For MALDI‐IMS analysis, age‐matched controls for Grade I‐III were obtained from the Netherlands.

### Classification of Disc Degeneration

The Thompson grading system was used to assess IVD degeneration, based on NP morphology, AF, and end plate intactness, and osteophyte formation. Thompson grading was employed for samples received from The Netherlands for the preliminary lectin histochemical‐based pilot study, where magnetic resonance imaging (MRI) images were not available to assess IVD degeneration using Pfirrmann grading. The Pfirrmann grading system was employed to assess the extent of disc degeneration, where grade varies from I to V. Sagittal T2‐weighted MR images of the lumbar spine were used to grade morphologic disc degeneration based on MR signal intensity, disc structure, the distinction between AF and NP, and disc height. This grading system was used for samples collected in Ireland that were subsequently used for *N*‐glycan analysis.

### *N*‐Glycan Analysis

*N*‐glycans were isolated from dried tissue homogenates immobilized in SDS‐PAGE gel blocks, enzymatically cleaved using PNGase F, labelled with 2‐aminobenzamide (2AB), and analyzed by Hydrophilic Interaction Liquid Chromatography (HILIC‐UPLC), Weak Anion Exchange (WAX‐UPLC), exoglycosidase digestions, and LC‐MS fluorescence methods. Detailed glycan isolation and analytical procedures are available in the supplementary methods. Formalin‐fixed paraffin‐embedded (FFPE) tissue sections were antigen‐retrieved, enzymatically digested with PNGase F, and analyzed by MALDI‐Fourier‐transform ion cyclotron resonance (FTICR) MS for high‐resolution mass spectrometry imaging. Glycan expression was statistically evaluated using SCiLS Lab 2024 software, normalized to the total ion current. Detailed experimental methods and parameters are available in the supplementary section. Full and detailed methods for sectioning of IVD tissue for histological examination and *N*‐glycan MALDI‐FTIMS/FTICR are available in the Supplementary material.

### Transcriptomics

Intervertebral disc tissues from degenerated (discectomy, Pfirrmann grade V) and healthy (adolescent idiopathic scoliosis, Pfirrmann grade I) patients were processed immediately following surgical procurement. Nucleus pulposus cells were isolated via Pronase and collagenase digestion, plated at 10 000 cells cm^−^^2^, and cultured under hypoxic conditions (1% O_2_, 5% CO_2_). Experiments were conducted using cells up to passage 4. Five experimental groups included healthy controls, degenerated controls, cytokine‐stimulated healthy NP cells, and Neu5Ac‐inhibitor‐treated cells (with or without cytokine stimulation). Cytokine treatment consisted of IL1β, IL‐6, and TNFα, refreshed after 48 h. The detailed methods are available in the Supplementary material.

### Statistical Analysis

All statistical analyses were conducted using GraphPad Prism 8 software unless otherwise stated. Pre‐processing included quality control, normalization to total ion current for glycan datasets, and evaluation of biological variance across replicates. UPLC and MALDI‐IMS data underwent z‐score normalization for comparison of glycan species. Data are presented as mean ± standard deviation (SD). Sample sizes (n) for each analysis are indicated in the figure legends. For comparisons between two groups, two‐sided Student's t‐tests were applied. For multiple group comparisons, one‐way or two‐way analysis of variance (ANOVA) was performed with Tukey's post hoc test for multiple comparisons. The significance threshold was set at α = 0.05, unless otherwise specified. Adjustments for multiple testing were applied using false discovery rate (FDR) correction where relevant (e.g., transcriptomic datasets). All assumptions for the applied tests were verified before use. Significance levels are represented in figures as *p < 0.05, **p < 0.01, ***p < 0.001, and ****p < 0.0001. Detailed statistical methods for each experiment are provided in the figure legends.

### Study Approval

Human tissues were collected from three hospitals in Galway: Galway University Hospital, Merlin Park Hospital, and Bon Secours Hospital, and from Our Lady's Children's Hospital, Crumlin, with ethical approval from NUI Galway Ethics Committee (CA 269), Health Service Executive, and Crumlin Research Ethics Committee. Collection of intervertebral disc specimens adhered to medical ethical regulations set out by University Medical Centre Utrecht (Utrecht, The Netherlands). All human samples included in this study were obtained with informed consent as per the ethical approval application.

## Conflict of Interest

The authors declare no conflict of interest.

## Author Contributions

K.J. and A.P. performed conceptualized. K.J., R.D., M.M.D., R.S., and A.P. carried out the methodology. K.J., A.S., and A.M.P. performed the investigation. A.D., M.M.D., R.O.F., R.S., and A.P. supervised the study. K.J. and A.S. wrote the original draft. K.J., A.S., M.M.D., R.O.F., R.S., and A.P. wrote, reviewed, and edited the final draft.

## Supporting information



Supporting Information

Supporting Information

## Data Availability

The data that support the findings of this study are available from the corresponding author upon reasonable request.
