# Supplementary material for: Inhibition of Hypersialylation in Human Intervertebral Disc Degeneration Modulates Inflammation and Metabolism
Source: Adv Sci (Weinh). 2025 Nov 14;13(6):e06669. doi: 10.1002/advs.202506669 (PMC12866799; doi:10.1002/advs.202506669)
Supplement: Supplementary file 1 — Supporting Information [file ADVS-13-e06669-s001.docx]

# Supplementary information text:

## Altered Nucleus Pulposus Glycosylation Motif Expression in Degeneration

The glycome is dynamic and responsive, present on protein, lipid, RNA and carbohydrate biomolecules through various conjugations. To investigate the presentation of glycosylated motifs in the IVD, lectin microarray was used to characterise the glycome in the human nucleus pulposus (NP) and annulus fibrosus (AF) in IVD degeneration (Supplementary dataset, Dataset S1). Strong binding was observed for a wide range of lectin targets for all samples, indicating abundant glycosylated motifs (Figure S1A-B). Total lectin binding was greatest in the health NP (NPH), indicating that this tissue was most highly glycosylated, yet sample glycosylation signatures are conserved across this sample set. Overall, principal component analysis revealed the greatest separation of NPH from all other samples, with healthy AF (AFH) separated from degenerated tissues (NPD and AFD) by PC2 (Figure S1C-F). The glycomic profile of healthy NP differed from AF, containing significantly more mannose, galactose, T-antigen and *N*-acetylgalactosamine motifs (p<0.05, Fig S1G-M). Galactose, T-antigen and *N*-acetylgalactosamine motifs were all decreased in the degenerated NP (p <0.05), and there was no significant difference in degenerated NP and AF, indicating the NP expresses a more AF-like glyco-phenotype in degeneration.

## Exploratory Analysis of the IVD Proteome

Interpreting changes in *N*-glycosylation requires characterisation of the proteome from which the *N*-glycans were cleaved, to account for non-enzymatic changes in glycosylation secondary to protein synthesis and degradation. Protein expression of the human IVD in degeneration was analysed by LC-MS/MS. Healthy tissue digests contained more proteins than degenerated tissues. A total of 1,590 proteins were identified across all samples. 427 of the 1,590 were verified across three or more samples per group. The relative expression of all proteins quantified was normalised to total protein content with intensity derived from the sum of all corresponding peptide intensities. A PERL script was employed to calculate theoretically observable peptides through *in silico* digestion to normalise protein intensities. Inclusion parameters for all tryptic peptides were set at 6-30 amino acids, while missed cleavages were excluded. The full list of all proteins with significantly altered expression is summarised in Dataset S4. The most abundantly expressed proteins consist of ECM constituents including glycoproteins, proteoglycans and collagens (Fig. S2E). In NP degeneration, 16 proteins (Top 5: IL17B, CLEC3A, MATN3, VIT, MPO) were significantly downregulated while 28 proteins were upregulated (Top 5: VTN, APOA4, C1QC, POSTN, APCS) vs healthy NP (Fig S2A). In AF degeneration, 11 proteins were significantly downregulated (Top 5: AOC, HAPLN3, CLEC3A, THBS4, IL17B) while 14 proteins were upregulated (Top 5: PLA2G2A, APOA4, APCS, TTR, TIMP3) vs healthy AF (Fig S2B).

PCA was performed to gain a global overview of the proteomic data on the complete set of filtered proteins. The first two principal components described a combined variance of 47.3%, with PC1 and PC2 contributing 29.7% and 17.6%, respectively (Fig. S2C-D). Clear demarcation was observed between healthy NP, healthy AF, and degenerated tissues. PC1 provides a strong separation of healthy and degenerated tissues.

Pathway analysis of the degenerated AF revealed an upregulation of complement activation (GO:0030449) and downregulation of collagen fibril organisation (GO:0030199), collagen biosynthesis and modifying enzymes (R-HSA-1650814). The degenerated NP revealed upregulation of complement cascade (R-HSA-977606), RAF/MAP kinase cascade (R-HSA-5673001), complement activation (GO:0030449), regulation of ERK1 and ERK2 cascade (GO:0070372); with subsequent downregulation of collagen biosynthesis and modifying enzymes (R-HSA-1650814), assembly of collagen fibrils and other multimeric structures (R-HSA-2022090), and skeletal system development (GO:0001501).

The Clustered Heatmap (Fig S2F) is a 2-way unsupervised hierarchical clustering technique that clusters the expression matrix along rows and columns, clustering similar genes and samples. The functional annotation of clusters demonstrates Hallmark collection pathway regulation for each gene module (Fig S2G-J). Gene modules S2 and S3 are associated with a degenerated proteome and have significant pathway annotation scores for the p53 pathway, PI3K Akt signalling, unfolded protein response and apoptosis. Gene modules S1 and S4 are associated with a healthy proteome and have positive annotation scores for oestrogen response, KRAS signalling, hypoxia and myogenesis.

### Supporting Materials and Methods Material and Reagents

AcroPrep™ Advance 96-filter plates and 10-kDa MWCO microcentrifuge filtration devices were purchased from Pall® Life Sciences, USA. Protogel™ was purchased from National Diagnostics™, France. 0.45 µm Millex-LH filters and C18 ziptips were purchased from Merck™, USA. 1 mL tuberculin BD Plastipak^©^ precision syringes were purchased from Medguard®, Ireland. Polypropylene 2 mL deep 96-well blocks and PhyNexus™ C18 phytip® columns were purchased from Fisher Scientific™, USA. Silverseal™ aluminium was purchased from Greiner Bio-One®, Austria. Plate seals were purchased from Cruinn Diagnostics®, Dublin. Sealing Mats were purchased from Phenomenex®, USA. Radio immunoprecipitation assay (RIPA) buffer, optimal cutting temperature (OCT) compound embedding medium and Superfrost plus slides were purchased from Thermo Fisher Scientific®, USA. Ammonium hydroxide solution was purchased from Honeywell Fluka™, USA. Leucine encephalin standard was purchased from Waters®, USA. All lectins were purchased from Vectors labs®, USA. PNGase F (P0709L) was purchased from New England Biolabs®, USA. All exoglycosidases were purchased from either New England Biolabs® or Prozyme®, USA. 3Fax-Peracetyl Neu5Ac was purchased from Merck. Human recombinant IL-1β, IL-6 and TNF-α were purchased from Peprotech. Primocin was purchased from InvivoGen. All other reagents were purchased from Merck®, USA, unless otherwise specified.

Sample processing and tissue homogenisation

Once all samples were collected for *N*-glycan analysis, the RIPA stored samples were thawed at 4°C. A stainless steel bead was added to each Eppendorf® and the tubes were added to the Qiagen Tissuelyser LT™ set at 50 Hz, for 80 minutes. If tissue was not completely homogenised after 80 min, homogenisation was continued for further 30 minutes. Once homogenisation was complete, Eppendorf tubes were spun at 16000 g for 20 minutes at 4°C. The supernatant was separated from the pellet and transferred to LoBind™ Eppendorf tube. The supernatant was dried in a vacuum centrifuge (Savant™ SPD131DDA SpeedVac™ Concentrator, ThermoFisher®) and stored at -80°C. This soluble fraction of tissue homogenate was further processed for *N*-glycan isolation.

## Lectin Microarray Analysis

The standard procedure used for the glycoprotein labelling: 50 µg by isolated glycoprotein of each sample (prior to gel block isolation in *N*-glycan analysis) was added to 10 µL of 10X phosphate buffer (500 mM, pH 8.3) and diluted to 99 µL with PBS. Afterwards, the solution was treated with 0.3 µL of Alexa-647 NHS ester (10 µg/µL in DMSO) for one hour at room temperature. The excess dye was quenched by the addition of 0.7 µL of 1 M Tris buffer. The fluorescently labelled glycoprotein solutions were not purified and were directly used in the lectin array analysis. Printed slides stored at -20°C were retrieved and quenched by immersion in a 50 mM ethanolamine solution in borate buffer (50 mM, pH 8.5) for 45 minutes at room temperature, and the quenched surface was then passivated by incubation in PBS containing 0.5% Tween-20, 0.4 mg/mL BSA, 1 mM CaCl_2_, 1 mM MgCl_2_ and 1 mM MnCl_2_ for 45 minutes at room temperature. The slide was dried by centrifugation. The glycoprotein samples were added to the corresponding wells and the incubation was carried out for 3.0 h at r.t. Samples were then aspirated, and the slide was washed with PBS for 5 minutes and dried by centrifugation and scanned. Microarray data interpretation: The images obtained from the G265BA microarray scanner (Agilent Technologies®) were analysed with Pro Scan Array Express software (PerkinElmer®) to determine fluorescence intensities. For each subarray, all fluorescence values were normalised to the highest single fluorescent value before combining lectin replicates to a single value. From six printed lectin spots per subarray, the maximum and minimum values were removed to generate an average from the median four values. Values from duplicate analyses for each sample were combined for the average and standard deviation values.

## *N*-Glycan Isolation – In Gel Block

Gels were made around the dried tissue homogenate to immobilise the *N*-glycosylated proteins made up of 64.7% Protogel, 32.30% Gel buffer (1.5M TRIS pH8.8) and 3% of 10% sodium dodecyl sulphate (SDS) solution. Gel volume was added until the homogenate was completely dissolved (100 μL for the sample). Ammonium peroxisulphate (APS) and *N,N,N,N’*-Tetramethyl-ethylenediamine (TEMED) were added to the gel solution at a ratio of 1:35. Gels were allowed to set for 20 minutes. They were then transferred to the freezer for 10 minutes for ease of cutting in the next step. Gels were chopped into 1 mm^3^ pieces on a clean glass plate using a clean scalpel. Gels were washed with 20 mM sodium bicarbonate solution and acetonitrile to wash impurities and unreacted gel components. The gels were reduced and alkylated using dithiothreitol (DTT, 0.5M) and iodoacetamide (IAA, 100 mM), respectively. This step removed disulphide bonds across peptides to expose *N*-glycan linkages to the peptide to be cleaved by PNGase F. The gel was further washed through dehydration and rehydration washes and PNGase F (1,250 units/mL) was added to each gel and incubated at 37°C overnight. Next, the glycans were eluted from the gels through several sonication steps and washes. The elution was filtered using a 0.45 μm LH Millipore filter and a 1 mL syringe and dried overnight in the vacuum centrifuge. Dried glycans were reduced in formic acid for 40 minutes, resuspended in 2-aminobenzamide (2AB) solution and incubated at 65 °C for 30 minutes for labelling through reductive amination (1). The solution was then transferred to Whatman 3MM chromatography paper and excess 2AB was washed away with acetonitrile (2). The glycans were eluted with water and dried in the vacuum centrifuge.

## Analysis of Human *N*-glycome on Hydrophilic Interaction Liquid Chromatography/ Ultra Performance Liquid Chromatography (HILIC-UPLC)

UPLC was performed using a BEH Glycan column (1.7 μm particles in 2.1x150 mm, Waters) on an Acquity UPLC equipped with a temperature control module and an Acquity fluorescence detector. Solvents A and B were composed of 50 mM formic acid adjusted to 4.4 pH with ammonia solution and acetonitrile (Sigma-Aldrich Acetonitrile E CHROMASOLV for HPLC, far UV), respectively. The column was maintained at a temperature of 40°C. Solvent A was applied using a linear gradient from 30-47% over 30 minutes followed by 47-70% A and finally 30% A to complete each run (2). Samples were prepared in 70% acetonitrile. The excitation wavelength was set at 330 nm with detection at 420 nm. Calibration was performed using hydrolysed and 2AB-labelled glucose oligomers as an external standard creating a dextran ladder that was used for every run (3).

## Weak Anion Exchange (WAX) – UPLC Determination of Sialylation

WAX – UPLC was completed using a Waters Acquity UPLC separations module complete with an Acquity HPLC fluorescence detector through the Empower Chromatography Workstation. The analytical column used was a Waters DEAE anion exchange column (75 x 7.5 mm, 10 µm particle size). Mobile phase A consisted of 20% v/v acetonitrile in water, (Milli-Q water, quality > 18.2 MΩ, TOC content < 5 ppb). Mobile phase B consisted of 0.1 M ammonium acetate buffer pH 7.0 in 20% v/v acetonitrile. A linear gradient of 0 to 5% solvent A over 12 minutes at a flow rate of 1 mL/min was applied, followed by 5−21% solvent A over 13 minutes and then 2−50% A over 25 minutes, 80−100% A over 5 minutes followed by five minutes at 100% (3). The standard used for charged state chromatogram annotation is 10% v/v fetuin N. Normal human serum (NHS) *N*-glycans labelled were used as an additional standard to ensure the instrument is correctly calibrated. Fluorescence detection was set at excitation/ emission wavelengths of *λ*_ex_ = 330 nm and *λ*_em_ = 420 nm, respectively.

## Exoglycosidase Digestions

Exoglycosidases were procured from Prozyme (San Leandro, CA) or New England Biolabs (AMF, BKF, GUH) (Hitchin, Herts, U.K.). The isolated 2AB-labelled glycans were digested for 18 hours at 37°C in 10 µL 50 mM sodium acetate buffer, pH 5.5 (except jack bean α-mannosidase (JBM) digestion which requires 100 mM sodium acetate, 2 mM Zn^2+^, pH 5.0). The following enzymes were used: almond meal α-fucosidase (AMF, EC 3.2.1.111), 40 mU/mL; *Arthrobacter ureafaciens* sialidase (ABS, EC 3.2.1.18), 0.5 U/mL; bovine kidney α-fucosidase (BKF, EC 3.2.1.51), 800 U/mL; bovine testes β-galactosidase (BTG, EC 3.2.1.23), 1 U/mL; β-*N*-acetylglucosaminidase cloned from *S. pneumonia*, expressed in *Escherichia coli* (GUH, EC 3.2.1.30), 8 U/mL (Prozyme) – 400 U/mL (NEB); Jack bean hexosaminidase (JBH, 3.2.1.52), 10 U/mL; Jack bean mannosidase (JBM, EC 3.2.1.24), 60 U/ mL; *Streptococcus pneumoniae* sialidase (NAN1, EC 3.2.1.18), 5 U/mL; *Streptococcus pneumoniae* β-galactosidase (SPG, EC 3.2.1.23), 0.4 U/mL. Glycosidases were removed after incubation by filtration through 10 kDa protein-binding EZ™ filters (Millipore Corporation). *N*-glycans were then analysed by UPLC as previously described (3).

## Liquid Chromatography-Mass Spectrometry-Fluorescence of *N*-glycans

Glycan profiles were obtained by negative ion nanoelectrospray LC-MS, performed by Acquity® UPLC system through a BEH Glycan Column (150 x 1.0 mm i.d., 1.7 µm particles), coupled to a Waters® Xevo® G2 QTOF system. The data acquisition was performed with the instrument set as previously described with augmentation (4). Data acquisition and analysis were performed using MassLynx™ (Waters®, Milford, MA, USA). The FLD excitation/emission spectra were set to 320 nm and 420 nm, respectively. The sample injection was 8 µL (75% MeCN). The flow rate was set to 0.15 mL/min and the column temperature was maintained at 60 °C. A linear gradient was applied as follows: 0.0 min 28% A 72% B, 1.0 min 28% A 72% B, 31.0 min 43% A 57% B, 32.0 min 45% A 55% B, 36.0 min 28% A 72% B, 40.0 min 28% A 72% B.

## Sectioning of IVD Tissue for Histological Examination

Discs from healthy and degenerated tissue were obtained from Utrecht University in the Netherlands. The IVD specimens were collected according to the medical ethical regulations (protocol 12-364) of the University Medical Centre Utrecht (Utrecht, The Netherlands). Isolated discs were fixed in 10% formalin for 48 hours, and decalcification of the vertebral bones was performed using Kristensen's decalcifying solution (18% (v/v) acetic acid/3.5% (w/v) sodium formate) at 4 °C. Following decalcification, samples were then washed in running tap water for 12 hours and transferred to 20% (w/v) sucrose solution until submerged at 4 °C. Optimal cutting temperature (OCT) compound was used to embed tissues, which were then snap-frozen in an isopentane bath with liquid nitrogen. Samples were stored at −80°C until sectioning at 10 µm on a cryostat (Leica CM1850).

## *N*-glycan MALDI-FTIMS/FTICR

Formalin-fixed paraffin-embedded (FFPE) tissues were sectioned at 5 µm and mounted onto charged slides (Superfrost™ Plus). Slides were subsequently dewaxed and rehydrated before undergoing antigen retrieval using citraconic anhydride buffer (25 µL citraconic anhydride, 2 µL HCl and 50 mL HPLC grade water, pH. 3.0-3.5) as previously described (5). After antigen retrieval, slides were desiccated and underwent digestion by recombinant PNGase F enzyme (Serva Electrophoresis GmbH, Heidelberg, Germany) applied using a TMSprayer™ (HTX Technologies LLC., Chapel Hill, NC). Enzyme solution was sprayed onto the slides at 25 µL/min for 15 passes at 45 °C. Slides were incubated in a humidified chamber for two hours at 37 °C. After incubation, slides were desiccated and 7 mg/mL CCA matrix was applied by the TMSprayer™ at 100 µL/min for 10 passes at 80 °C. Slides were stored in a desiccator overnight until further analysis by MALDI-FTICR MS. Released *N-*glycan ions were detected using a MALDI FTICR scimaX™ (Bruker Daltonics, Germany) for accurate, high resolution mass analysis, operating in positive ion mode with a Smart Beam II laser operating at 1000 Hz and a laser spot size of 20 µm. The signal was collected at a raster width of 200 µm between spots. A total of 10 laser shots were collected to form each pixel, calibrated to release a total of 5× 10^8^ ions from each spot. Following the acquisition, data was processed and images of expressed glycans were generated using SCiLS™ Lab 2024 software (Bruker Daltonics, Germany), where ions in the range of 650-3400 m/z were analysed and statistically evaluated the expression across sample cohorts normalised to the total ion count. Observed mass/ charge ratios were searched against glycan databases using GlycoWorkBench (6). Represented glycan structures were generated in GlycoWorkBench and composition was determined by accurate mass (<10 ppm error), and previous structural characterisation elucidated by UPLC for the human *N-*glycan profile described above. SCiLS 2018 (Bruker Daltonics) imaging software was used to further analyse glycan expression and statistically evaluate expression across sample cohorts normalised to the total ion current.

## Histopathological overview

After MALDI analysis, the matrix was removed from the slides by dipping in 100% acetone until slides were clear. Slides were then washed in demineralised water for five minutes before being subjected to Weigert’s Haematoxylin for five minutes. Slides were then washed in running tap water for 10 minutes, rinsed in distilled water and counterstained with filtered 0.4% Fast Green for four minutes, subjected to 1% acetic acid two times for a total of five minutes and stained with 0.125% aqueous Safranin for five minutes. The sections were then dehydrated in 96% Ethanol for one minute twice, followed by 100% ethanol for five minutes and xylene two times for five minutes and mounted using DPX mountant.

## Proteomic Evaluation of Human IVD

After *N*-glycans had been released during glycan isolation, the gels were rehydrated, washed and dried in a vacuum centrifuge. 200 µL of trypsin in 50 mM ammonium bicarbonate (ratio enzyme: substrate 1:50) was added to each sample and incubated at 37 °C overnight. The digested peptides were eluted with 1% formic acid 50% AcN: H_2_O through multiple washes and sonication steps. Trifluoroacetic acid (TFA) was added to the sample to a final concentration of 0.1% before purification with a C18 Zip tip. Samples were dried to approx. 10-20 µL volume and frozen at -20 °C until LC-MS analysis. Samples were run on a Q Exactive™ Hybrid Quadrupole-Orbitrap™ Mass Spectrometer (UCD Conway, Dublin). Briefly, samples were dissolved in 0.1% formic acid, loaded onto a fused silica emitter (75 µm ø), pulled with a laser puller and packed with reverse-phase media. An increasing acetonitrile gradient was applied over 47 minutes at a 250 nL/min flow rate. The instrument was operated in positive-ion mode with a potential of 2,300 V applied to the frit and a capillary temperature of 320°C. A high-resolution (70,000) MS scan across 300–1,600 m/z was performed with Q Exactive to identify the eight most intense ions, followed by MS/MS analysis with higher-energy collisional dissociation. Protein identification was performed by searching the raw data against the *Homo sapiens* (Human) subset of the UniProt Swiss-Prot database (UP000005640_9606.fasta) using MaxQuant computational platform (Max-Planck-institute of Biochemistry). Label-free quantification was performed based on specified peptides with specified enzymatic cleavage for Trypsin/P with fixed modification of carboxymethylation and deamidation, as outlined previously (7). Each peptide used for protein quantification was subject to FDR filtering of <1% to be accepted for analysis. MaxQuant data was exported to Perseus® software for proteomic analysis (8). The output of differentially expressed proteins was further investigated using Ingenuity Pathway Analysis® (IPA; Qiagen®, Redwood City, USA). Data was also independently analysed using PEAKS studio (Bioinformatics Solutions® Inc.) for peptide identification and label-free quantification to validate MaxQuant identification and quantification.

## Transcriptomics

Degenerated cells (discectomy patients, Pfirrmann grade V) and healthy cells (adolescent idiopathic scoliosis, Pfirrmann grade I) were procured intraoperatively with consent. AF and NP were identified and separated in the theatre by the surgeon. The tissue was weighed based upon an addition to a pre-weighed falcon tube containing PBS (pH 7.4) and 1 % P/S. After washing, the tissue was minced and a 0.2 % Pronase solution (pH 7.4) was added for 1 h at 37 °C on a shaker plate at 300 rpm. The tissue was washed twice with PBS and incubated in collagenase solution (100 U/mL collagenase in α-MEM and 10 % FBS) (pH 7.4). The sample was digested overnight and filtered through a 100 µm cell strainer. The cell solution was centrifuged and washed before cell counting. Cells were plated at a density of 10,000 cells/cm^2^. Cells were cultured in a complete medium containing α-MEM, 10 % FBS and 1 % P/S in hypoxia (1% oxygen, 5% CO_2_) at 37 °C. All experiments were performed on cells within passage 4 or sooner due to the loss of cell phenotype in 2D culture conditions (9). Healthy Control (H-CON: NP cells extracted from healthy IVD), Degenerated Control (D-CON: NP cells extracted from degenerated IVD), Healthy Cytokine (H-CYTKN: healthy NP cells stimulated with cytokine cocktail – IL1β, IL-6, TNFα), Healthy Treated (H-TREAT: combination of healthy NP cells, cytokine cocktail and Neu5Ac-inhib), Degenerated Treated (D-TREAT: degenerated NP cells and Neu5Ac-inhib). The cytokine-containing medium was refreshed at 48 h. The ‘Degenerated Treated’ group does not contain the cytokine cocktail, as these cells were hypothesised to be preconditioned having already been exposed to an inflammatory microenvironment in vivo.

## RNA Sequencing

The compressed paired-end human mRNA-seq data in fastq format over 80 gigabytes from Illumina PE150. The ‘fastqc’ v.3.2 quality control tool (Babraham Bioinformatics) was applied. Each group consisted of three biological replicates (five experimental groups), and each biological replicate was divided into two paired-end files. The "multiqc" tool v.2.1.4 generated duplication reads, average GC content, and entire sequences but also sequence quality histograms, per sequence quality scores, per sequence, quality content and adapter content graphs (10). ‘trim-galore v.4.2’ performed adapter trimming and low-quality read filtering. Moreover, 'fastp' tools v.2.1 were used for filtering low-quality cells (11). After completing these steps, each replicate has over 99 % of the read-passing filter. Alignment, assembly and quantification were performed using Kallisto" tools v.2.3. (ENSEMBL Human cDNA file (the transcriptFASTAfile: Homo_sapiens.GRCh38). We applied the "tximport" package to import transcript-level abundances from Kallisto quantification tools into R studio and convert into gene counts for downstream analysis. The biomart "ensembl" database and the "hsapiens_gene_ensembl" dataset were used for connecting and mapping transcript IDs to gene IDs.

"DESeq2" v.1.36.0 (12) was used to perform differential expression analysis. We explored four different experiment data analyses between two groups: H-CYTKN vs H-CON, D-CON vs H-CON, H-TREAT vs H-CYTKN and D-TREAT vs D-CON. A PCA plot that identifies the samples as clusters or patterns and relationships in the data was generated through the PCAtools R package. In the differential gene analysis, the adjusted p-value cutoff (FDR) and alpha value were 0.05, the Benjamini-Hochberg method was applied for adjusting p-values, and independent hypothesis weighting was chosen as the performance of independent filtering through IHW v.1.24.0 Bioconductor package (13). A shrinkage method called "Adaptive Permutation based Empirical Bayes Gene-wise Linear Models" known as 'apeglm' (14) were applied to account for unreliably large log fold change estimates. Biological and statistically significant differentially expressed upregulated and downregulated genes are determined by log_2_FC >1 and adjusted *p*-value <0.05. Different gene set enrichment in GO, KEGG and Reactome pathways was analysed using "fgsea" v.1.24.0 R package with default parameters (normalised enrichment score - <2.2 />-2.2, FDR 0.05). The results are described in dot plot and the heatmap shows the differentially expressed genes in each specific enriched gene set. Finally, the over-representation methods of "clusterprofiler package" v.4.8.1 (15) were utilised to identify functionally enriched pathways with parameters of 0.05 adjusted p-value and q-value cut-off and the Benjamini-Hochberg method as pAdjustmethod after changing gene-IDs into Entrez-IDs, producing dot plots and category plots.

References

1. J. C. Bigge, *et al.*, Nonselective and efficient fluorescent labeling of glycans using 2-amino benzamide and anthranilic acid. *Anal. Biochem.* **230**, 229–238 (1995).

2. L. Royle, *et al.*, HPLC-based analysis of serum N-glycans on a 96-well plate platform with dedicated database software. *Anal. Biochem.* **376**, 1–12 (2008).

3. L. Royle, C. M. Radcliffe, R. A. Dwek, P. M. Rudd, “Detailed structural analysis of N-glycans released from glycoproteins in SDS-PAGE gel bands using HPLC combined with exoglycosidase array digestions” in *Glycobiology Protocols*, (Humana Press), pp. 125–144.

4. H. Stöckmann, R. M. Duke, S. Millán Martín, P. M. Rudd, Ultrahigh throughput, ultrafiltration-based N-glycomics platform for ultraperformance liquid chromatography (ULTRA3). *Anal. Chem.* **87**, 8316–8322 (2015).

5. A. L. Rebelo, *et al.*, Complete spatial characterisation of N-glycosylation upon striatal neuroinflammation in the rodent brain. *J. Neuroinflammation* **18**, 1–19 (2021).

6. D. Damerell, *et al.*, “Annotation of glycomics MS and MS/MS spectra using the GlycoWorkbench software tool” in *Glycoinformatics*, (Springer New York, 2015), pp. 3–15.

7. J. Cox, *et al.*, A practical guide to the maxquant computational platform for silac-based quantitative proteomics. *Nat. Protoc.* **4**, 698–705 (2009).

8. S. Tyanova, J. Cox, “Perseus: A bioinformatics platform for integrative analysis of proteomics data in cancer research” in *Methods in Molecular Biology*, (2018).

9. G. Gao, *et al.*, Naringin Protects Against Interleukin 1β (IL-1β)-Induced Human Nucleus Pulposus Cells Degeneration via Downregulation Nuclear Factor kappa B (NF-κB) Pathway and p53 Expression. *Med. Sci. Monit.* **25**, 9963 (2019).

10. P. Ewels, M. Magnusson, S. Lundin, M. Käller, MultiQC: summarize analysis results for multiple tools and samples in a single report. *Bioinformatics* **32**, 3047–3048 (2016).

11. S. Chen, Y. Zhou, Y. Chen, J. Gu, fastp: an ultra-fast all-in-one FASTQ preprocessor. *Bioinformatics* **34**, i884–i890 (2018).

12. M. I. Love, W. Huber, S. Anders, Moderated estimation of fold change and dispersion for RNA-seq data with DESeq2. *Genome Biol.* **15** (2014).

13. N. Ignatiadis, B. Klaus, J. B. Zaugg, W. Huber, Data-driven hypothesis weighting increases detection power in genome-scale multiple testing. *Nat. Methods* **13**, 577–580 (2016).

14. A. Zhu, J. G. Ibrahim, M. I. Love, Heavy-tailed prior distributions for sequence count data: removing the noise and preserving large differences. *Bioinformatics* **35**, 2084–2092 (2018).

15. G. Yu, L.-G. Wang, Y. Han, Q.-Y. He, clusterProfiler: an R package for comparing biological themes among gene clusters. *Omi. A J. Integr. Biol.* **16**, 284–287 (2012).

**Figure S1. Glycosylation is downregulated in IVD degeneration.** A) Fluorescence intensity of lectin binding in healthy NP (NPH), healthy AF (AFH), degenerated NP (NPD), and degenerated AF (AFH). B) Heatmap of z-score normalised lectin expression. C+D) Scatterplot of fluorescent intensities values for NP (C) and AF (D. E+F) Principal component analysis of lectin fluorescent intensities for each group (E) and loadings demonstrating individual loading contribution (F). G-M) Box plots of cumulative glycosylation motif expression represented as combined z-score normalised lectin expression. ANOVA was performed, followed by Tukey’s post hoc comparison. *p <0.05, **p <0.01, ***p <0.001.

**Figure S2. IVD degeneration is associated with acute phase signalling and apoptosis.** (A+B) Volcano plots of significantly differentially expressed proteins across the NP (A) and AF (B) in degeneration, two-sided t-test, FDR <0.05. C+D) Principal component analysis and loadings separating healthy and degenerated IVD samples. E) Heatmap of protein’s relative abundance across IVD samples - top 20 most highly expressed proteins. F) Subunit separation based on experimental groups. G-J) Most highly activated pathways in each subunit.

**Figure S3.** A+B) Principal component analysis and loadings demonstrating glycan trait contributions. C-E) Principal component analysis loadings with highlighted traits; mannosylated (green) and lactoaminylated (blue) glycans (K), Tetraantennary (pink) and biantennary (brown) (L), and outer arm fucosylated (red) and alpha-2,6 sialylated (purple) (M). Two-way ANOVA, Tukey’s post hoc test, *p <0.05, ***p <0.001

**Figure S4. Transcriptional data from human NP cells under inflammatory and glycosylation inhibitor treated conditions.** A+B) Hallmark pathway regulation demonstrating significantly dysregulated pathways in cytokine and cytokine/Neu5Ac-inhib conditions (H-TREAT vs H-CYTKN and H-CYTKN vs H-CON) (A) and unstimulated/Neu5Ac-inhib treated cells (D-TREAT vs D-CON and D-CON vs H-CON) (B), false discovery rates (FDR) <0.2. C) Significantly downregulated genes in the inflammatory response in Neu5Ac-inhib treated degenerated NP cells, p <0.05. D-F) Significantly dysregulated genes in TNFA signalling (D), Inflammatory response (E) and Epithelial Mesenchymal Transition (F) in Neu5Ac-inhib treated cytokine-stimulated NP cells, p <0.05.

**Table S1.** Summary of identified N-glycans from human IVD - Percentage areas form UPLC chromatograms. Structural abbreviations denoting glycan structure are as follows: all N-glycans have a GlcNAc(2)Man(3)/M3 core; F(6) indicates a α1,6-linked core-fucose; Mx - number (x) of mannose on core GlcNAcs; Ax, number of antenna (GlcNAc) on trimannosyl core; A2, biantennary with β1,2-linked GlcNAcs; A3, triantennary with two β1,2-linked GlcNAcs and a third β1,4-linked GlcNAc; A4, tetraantennary with two β1,2-linked GlcNAcs, a β1,4-linked GlcNAc and a β1,6-linked GlcNAc; B, bisecting β1,4-linked GlcNAc; Gx, number (x) of β1,3 or β1,4-linked galactose on antenna (linkage indicated by closed brackets); Fx, number (x) of α1,3 linked fucose to antenna GlcNAc; Sx, number (x) of terminal sialic acids linked to galactose (linkage indicated by closed brackets); Lacx, number (x) of lactosamine (Gal-β1,4-GlcNAc) structures; Ac – acetylation; Sul – sulphation; Glc – glucose; jnk denotes non-glycan material.

| **Peak number** | **Glycans** | **AF healthy** | | **NP healthy** | | **AF degenerated** | | **NP degenerated** | |
| --- | --- | --- | --- | --- | --- | --- | --- | --- | --- |
|  |  | **GU** | Glycan % Area | **GU** | Glycan % Area | **GU** | Glycan % Area | **GU** | Glycan % Area |
| **1** | M3 | **4.16** | **0.36** | **4.14** | **0.45** | **4.18** | **0.32** | **4.16** | **0.42** |
|  |  | **4.28** |  | **4.26** |  | **4.29** |  | **4.28** |  |
| **2** | F(6)M3 | **4.76** | 0.04 | **4.73** | **0.25** | **4.78** | **0.35** | **4.75** | **0.45** |
|  | M3B |  | **0.16** |  | 0.18 |  | 0.15 |  | 0.20 |
|  | A1 |  | 0.07 |  | 0.05 | **4.84** | 0.07 | **4.81** | 0.20 |
| **3** | jnk | **4.93** | **0.30** | **4.91** | **0.22** | **4.95** | **0.09** | **4.92** | **0.12** |
| **4** | M4 | **5.11** | **0.45** | **5.09** | **0.86** | **5.14** | **0.50** | **5.11** | **0.68** |
| **5** | A1B | **5.32** | **0.15** | **5.29** | **0.30** | **5.35** | 0.12 | **5.31** | 0.10 |
|  | F(6)A1 |  | 0.06 |  | 0.06 |  | **0.30** |  | **0.50** |
|  | A2 |  | 0.07 |  | 0.05 |  | **0.30** |  | 0.25 |
| **6** | jnk | **5.67** | **0.31** | **5.65** | **0.26** | **5.71** | **0.15** | **5.67** | **0.20** |
| **7** | A1G(3)1 | **5.84** | 0.05 | **5.83** | 0.10 | **5.90** | 0.05 | **5.85** | 0.05 |
|  | A2B |  | 0.04 |  | 0.05 |  | 0.05 |  | 0.05 |
|  | M4A1 |  | 0.05 |  | 0.05 |  | 0.20 |  | 0.05 |
|  | F(6)A2 |  | **0.25** |  | **0.25** |  | **0.55** |  | **0.25** |
|  | A3 |  | 0.05 |  | 0.05 |  | 0.05 |  | 0.05 |
| **8** | F(6)M4A1 | **6.19** | 0.90 | **6.17** | 0.50 | **6.23** | 0.90 | **6.19** | 0.90 |
|  | F(6)A1G1(3) |  | 0.75 |  | 0.75 |  | 0.50 |  | 0.30 |
|  | M5 |  | **9.95** |  | **13.44** |  | **10.62** |  | **10.81** |
|  | A3B |  | 0.35 |  | 0.60 |  | 0.30 |  | 0.35 |
|  | F(6)A2B |  | 0.10 |  | 0.10 |  | 0.10 |  | 0.10 |
|  | A2G(3)1 |  | 0.35 |  | 0.35 |  | 0.30 |  | 0.10 |
|  | A1G(3)1S(3)1 |  | 0.70 |  | 0.05 |  | 0.01 |  | 0.35 |
|  | F(6)A3 |  | 0.60 |  | 0.60 |  | 0.30 |  | 0.60 |
|  | M4A2 |  | 0.05 |  | 0.05 |  | 0.05 |  | 0.10 |
| **9** | F(6)A3B | **6.69** | 0.01 | **6.67** | 0.01 | **6.74** | 0.01 | **6.69** | 0.01 |
|  | A4 |  | 0.17 |  | 0.10 |  | 0.24 |  | 0.25 |
|  | A1G(3)1S(6)1 |  | 0.10 |  | 0.10 |  | 0.01 |  | 0.01 |
|  | A3F1 |  | 0.06 |  | 0.05 |  | 0.08 |  | 0.05 |
|  | A2BG(4)1 |  | 0.01 |  | 0.01 |  | 0.01 |  | 0.10 |
|  | M4A1G(3)1 |  | 0.20 |  | 0.05 |  | 0.05 |  | 0.20 |
|  | M5A1 |  | **0.40** |  | **0.15** |  | **0.40** |  | **0.40** |
|  | F(6)A2[6]G(4)1 |  | 0.15 |  | **0.15** |  | 0.13 |  | 0.25 |
| **10** | F(6)A2F1 | **6.80** | 0.20 | **6.79** | 0.10 | **6.86** | 0.10 | **6.82** | 0.09 |
|  | F(6)A2[3]G(4)1 |  | **0.86** |  | **0.75** |  | **0.85** |  | **1.00** |
|  | F(6)A2[3]G(3)1 |  | 0.12 |  | 0.10 |  | 0.20 |  | 0.30 |
| **11** | A3BG(4)1 | **6.96** | 0.02 | **6.95** | 0.05 | **7.01** | 0.20 | **6.96** | 0.05 |
|  | A4B |  | 0.03 |  | 0.01 |  | 0.01 |  | 0.05 |
|  | F(6)A2GalNAc2 |  | 0.15 |  | **0.15** |  | **0.40** |  | 0.15 |
|  | F(6)A4 |  | **0.24** |  | 0.10 |  | 0.09 |  | **0.25** |
| **12** | M6 | **7.10** | **4.53** | **7.09** | **4.08** | **7.15** | **2.84** | **7.11** | **2.23** |
|  | A2G(4)1S(3)1 |  | 0.50 |  | 0.50 |  | 0.34 |  | 0.20 |
|  | F(6)A3G(3)1 |  | 0.50 |  | 0.50 |  | 0.40 |  | 0.50 |
|  | A2F1G1 |  | 0.20 |  | 0.10 |  | 0.18 |  | 0.20 |
| **13** | M4A2G(4)1 | **7.25** | 0.63 | **7.24** | 0.40 | **7.30** | **0.60** | **7.26** | 0.60 |
|  | A2G1S1Ac |  | **0.73** |  | **0.75** |  | 0.40 |  | 0.45 |
|  | F(6)A3F1 |  | 0.40 |  | 0.45 |  | 0.10 |  | 0.60 |
|  | A2G(4,4)2 |  | 0.30 |  | 0.15 |  | **0.60** |  | **0.80** |
|  | F(6)A1GalNAc1S(6)1 |  | 0.05 |  | 0.01 |  | 0.01 |  | 0.01 |
|  | F(6)A2G1GalNAc1 |  | 0.01 |  | 0.05 |  | **0.60** |  | 0.20 |
|  | A2BG(4)1S(3)1 |  | 0.01 |  | 0.01 |  | 0.01 |  | 0.01 |
|  | A4F1 |  | 0.03 |  | 0.01 |  | 0.21 |  | 0.10 |
| **14** | F(6)A1G(3)1S(6)1 | **7.60** | 0.01 | **7.59** | 0.01 | **7.65** | 0.01 | **7.60** | 0.30 |
|  | F(6)A1G1Lac1 |  | 1.70 |  | 1.50 |  | 2.00 |  | 2.00 |
|  | M4A1G1S(3)1 |  | 0.01 |  | 0.01 |  | 0.01 |  | 0.01 |
|  | F(6)A2F1G1 |  | 0.10 |  | 0.05 |  | 0.30 |  | 0.30 |
|  | F(6)A2G(4,4)2 |  | **2.60** |  | **2.90** |  | **2.90** |  | **2.80** |
|  | A2BG(4)1S(6)1 |  | 0.01 |  | 0.01 |  | 0.01 |  | 0.01 |
| **15** | F(6)A2BG(4)1S(3)1 | **7.74** | 0.02 | **7.72** | 0.01 | **7.79** | 0.01 | **7.74** | 0.01 |
|  | F(6)A2[3]G(4)1S(3)1 |  | 0.02 |  | 0.01 |  | 0.21 |  | 0.20 |
|  | F(6)A2GalNAc1S(6)1 |  | 0.02 |  | 0.01 |  | 0.01 |  | 0.01 |
|  | F(6)A2F1GalNAc2 |  | 0.75 |  | 0.50 |  | **0.90** |  | **1.00** |
|  | A4BG(4)1 |  | 0.20 |  | 0.01 |  | 0.01 |  | 0.01 |
|  | F(6)A2BG(4,4)2 |  | 0.10 |  | 0.15 |  | 0.01 |  | 0.10 |
|  | A2G(4)1S(6)1 |  | 0.02 |  | 0.01 |  | 0.70 |  | 0.70 |
|  | F(6)A3G(4,4)2 |  | **1.10** |  | **0.70** |  | 0.40 |  | 0.80 |
| **16** | F(6)A4F1 | **8.03** | 0.03 | **8.01** | 0.01 | **8.08** | 0.40 | **8.03** | 0.30 |
|  | F(6)A2F1GalNAc1S(3)1 |  | 0.65 |  | 0.01 |  | 0.01 |  | 0.25 |
|  | A2G1GalNAc1S(6)1 |  | 0.80 |  | 0.60 |  | 0.70 |  | 0.80 |
|  | F(6)A3F1G1 |  | 0.90 |  | 0.50 |  | 0.90 |  | 0.46 |
|  | M7D1 |  | **1.10** |  | 0.73 |  | 0.80 |  | 0.73 |
|  | A2F1G(3)2 |  | 0.60 |  | 0.50 |  | 0.30 |  | 0.20 |
|  | F(6)A2G1G(3Sul)1 |  | 0.60 |  | 0.01 |  | **1.00** |  | 0.55 |
|  | F(6)A2G(3Sul)1S(3)1 |  | 0.32 |  | 0.20 |  | 0.40 |  | 0.10 |
|  | A2G(4)2S(3)1(9Ac) |  | 0.70 |  | 0.25 |  | 0.70 |  | 1.00 |
|  | A2G(4,4)2S(3)1 |  | 0.30 |  | 0.25 |  | 0.60 |  | 0.70 |
|  | F(6)A2F1G1GalNAc1 |  | 0.50 |  | **1.50** |  | **1.00** |  | **1.30** |
|  | F(6)M4A1G(4)1S(3)1 |  | 0.15 |  | 0.20 |  | 0.40 |  | 0.30 |
|  | M4A1G(4)1S(6)1 |  | 0.00 |  | 0.00 |  | 0.10 |  | 0.10 |
|  | F(6)A2[3]G(4)1S(6)1 |  | 0.20 |  | 0.25 |  | 0.10 |  | 0.30 |
| **17** | F(6)A2[3]G(4)1S(6)1 | **8.19** | 0.01 | **8.17** | **0.01** | **8.23** | 0.01 | **8.19** | 0.00 |
|  | A2BG(4,4)2(3)1 |  | 0.01 |  | **0.01** |  | **0.11** |  | **0.01** |
|  | F(6)A2BG(4)1S(6)1 |  | 0.01 |  | **0.01** |  | 0.01 |  | **0.01** |
|  | F(6)A2GalNAc2S(6)1 |  | **0.02** |  | **0.01** |  | 0.01 |  | **0.01** |
| **18** | A4F1G1 | **8.37** | 0.12 | **8.35** | 0.20 | **8.41** | 0.50 | **8.37** | 0.30 |
|  | A3F1G(4,4)2 |  | 0.30 |  | 0.25 |  | 0.50 |  | 0.30 |
|  | A2G1GalNAc1S(3,6)2 |  | 0.00 |  | 0.00 |  | 0.20 |  | 0.00 |
|  | F(6)A2G(3Sul)1S(6)1 |  | 0.01 |  | 0.01 |  | 0.01 |  | 0.05 |
|  | F(6)A2F1GalNAc1S(6)1 |  | 0.50 |  | 0.20 |  | 0.50 |  | 0.95 |
|  | F(6)A2G(4,4)2S(3)1 |  | **1.50** |  | **2.40** |  | **1.40** |  | **2.10** |
|  | F(6)M4A1G(4)1S(6)1 |  | 1.20 |  | 0.90 |  | 0.90 |  | 0.90 |
|  | F(6)A3G(3)1S(6)1 |  | 0.40 |  | 0.11 |  | 0.10 |  | 0.10 |
|  | A3G(3,3,3)3 |  | 0.67 |  | 0.50 |  | 1.10 |  | 0.80 |
|  | A2G(4,4)2S(6)1 |  | 0.80 |  | 0.75 |  | 1.00 |  | 1.00 |
|  | F(6)A2F1G(3)2 |  | 0.42 |  | 0.30 |  | 0.40 |  | 0.40 |
|  | A3BG(4,4,4)3 |  | 1.00 |  | 0.80 |  | 1.00 |  | 1.30 |
| **19** | F(6)A2F1GalNAc2S(6)1 | **8.49** | 0.02 | **8.47** | 0.01 | **8.52** | 0.01 | **8.47** | 0.01 |
|  | A4BG(4)1S(3)1 |  | 0.01 |  | 0.01 |  | 0.01 |  | 0.01 |
|  | A4BF1G1 |  | **0.20** |  | **0.05** |  | **0.20** |  | **0.30** |
| **20** | F(6)A2F2GalNAc2 | **8.60** | **0.63** | **8.59** | **0.75** | **8.65** | **0.70** | **8.60** | **0.50** |
|  | F(6)A4F2 |  | 0.48 |  | 0.25 |  | 0.40 |  | 0.35 |
|  | A3BG(4,4)2S(3)1 |  | 0.10 |  | 0.01 |  | 0.01 |  | 0.01 |
|  | F(6)A2BG(4,4)2S(3)1 |  | 0.10 |  | 0.01 |  | 0.01 |  | 0.01 |
|  | F(6)A4G(3,3)2 |  | 0.20 |  | 0.20 |  | 0.35 |  | 0.45 |
|  | F(6)A3F1G1S(3)1 |  | 0.05 |  | 0.01 |  | 0.50 |  | 0.01 |
|  | F(6)A2G1GalNAc1S(6)1 |  | 0.20 |  | 0.40 |  | 0.01 |  | 0.30 |
|  | A2BG(4,4)2S(6)1 |  | 0.01 |  | 0.01 |  | 0.01 |  | 0.01 |
| **21** | F(6)A3G(3,4,4)3 | **8.79** | 1.50 | **8.78** | 1.50 | **8.83** | 1.90 | **8.79** | 1.15 |
|  | F(6)A2G1G(3Sul)1S(3)1 |  | 0.01 |  | 0.01 |  | 0.01 |  | 0.05 |
|  | A4BG(4)1S(6)1 |  | 0.50 |  | 0.10 |  | 0.30 |  | 0.50 |
|  | M8 |  | 0.95 |  | 0.40 |  | 0.25 |  | 0.31 |
|  | F(6)A2G1GalNAc(Sul)1S(6)1 |  | 0.60 |  | 2.00 |  | 1.40 |  | 0.68 |
|  | F(6)A2GalNAc2sS(3,6)1 |  | 0.05 |  | 0.60 |  | 0.60 |  | 0.40 |
|  | F(6)A2G(4,4)2S(6)1 |  | **8.60** |  | **6.80** |  | **7.80** |  | **8.08** |
|  | F(6)A4BG1S(3)1 |  | 0.30 |  | 0.10 |  | 0.30 |  | 0.30 |
| **22** | F(6)A2G(4,4)2S(6)1 | **8.94** | 0.01 | **8.93** | 0.01 | **8.99** | 0.01 | **8.95** | 0.01 |
|  | M8 |  | 0.34 |  | 0.34 |  | 0.38 |  | 0.25 |
|  | F(6)A2F1GalNAc2S(6)1 |  | 0.01 |  | 0.01 |  | 0.01 |  | 0.01 |
|  | A2BG(4,4)2S(3,3)2 |  | 0.01 |  | 0.01 |  | 0.01 |  | 0.01 |
|  | F(6)A2BG(4,4)2S(6)1 |  | 0.01 |  | 0.01 |  | 0.01 |  | 0.01 |
|  | A3BG(4,4)2S(6)1 |  | 0.01 |  | 0.01 |  | 0.01 |  | 0.01 |
|  | F(6)A3G(4,4,4)3 |  | **3.00** |  | **2.20** |  | **2.30** |  | **3.20** |
| **23** | F(6)A3G(4,4,4)3 | **9.15** | 0.11 | **9.14** | 0.50 | **9.20** | **0.90** | **9.15** | **0.90** |
|  | F(6)A2G1G(3Sul)1S(6)1 |  | 0.20 |  | 0.05 |  | 0.20 |  | 0.17 |
|  | A4F1G1S(3)1 |  | 0.05 |  | 0.05 |  | 0.05 |  | 0.60 |
|  | A3BG(4,4)2S(3,3)2 |  | **0.50** |  | **0.40** |  | 0.50 |  | 0.40 |
|  | F(6)A2BG(4,4)2S(3,3)2 |  | 0.21 |  | 0.35 |  | 0.20 |  | 0.01 |
|  | A3F1G(4)2S(3)1 |  | 0.05 |  | 0.25 |  | 0.05 |  | 0.10 |
| **24** | F(6)A3F1G1S(6)1 | **9.36** | 0.01 | **9.35** | 0.01 | **9.41** | 0.01 | **9.37** | 0.01 |
|  | A2BG(4,4)2S(3,6)2 |  | 0.01 |  | 0.01 |  | 0.01 |  | 0.01 |
|  | F(6)A3F1G(4)2GalNAc1 |  | 0.40 |  | **0.60** |  | 0.30 |  | 0.20 |
|  | F(6)A2G1GalNAc1S(3,6)2 |  | 0.01 |  | 0.01 |  | 0.05 |  | 0.01 |
|  | F(6)A2G(4,4)2S(3,3)2 |  | 0.01 |  | 0.01 |  | 0.05 |  | 0.20 |
|  | A2G(4,4)2S(3,6)2 |  | 0.02 |  | 0.01 |  | 0.30 |  | 0.25 |
|  | A4BG(4,4)2S(3)1 |  | 0.01 |  | 0.01 |  | 0.01 |  | 0.01 |
|  | A3G(4,4)3S(9Ac)1 |  | **0.60** |  | 0.25 |  | **0.60** |  | **0.55** |
|  | F(6)A2F1G1GalNAc1S(6)1 |  | 0.02 |  | 0.01 |  | **0.60** |  | 0.01 |
|  | F(6)A4BG(4)1S(6)1 |  | 0.02 |  | 0.01 |  | 0.05 |  | 0.10 |
|  | A3F1G(4)2S(6)1 |  | 0.02 |  | 0.01 |  | 0.01 |  | 0.01 |
|  | F(6)A4G(3,3)2S(3)1 |  | 0.02 |  | 0.01 |  | 0.05 |  | 0.10 |
| **25** | M9 | **9.58** | 0.87 | **9.58** | 0.59 | **9.67** | 0.76 | **9.64** | 0.80 |
|  | F(6)A2G(4,4)2S(3,6)2 |  | 1.00 |  | **1.50** |  | 1.00 |  | 0.80 |
|  | A4BF1G1S(3)1 |  | 0.30 |  | 0.20 |  | 0.10 |  | 0.30 |
|  | F(6)A2G1GalNAc1S(6,6)2 |  | 0.62 |  | 0.70 |  | 0.40 |  | 0.50 |
|  | F(6)A3G(4)2GalNAc1S(3)1 |  | **1.50** |  | 1.20 |  | 1.10 |  | 0.80 |
|  | A3BG(4,4,4)3S(3)1 |  | 0.35 |  | 0.30 |  | 0.30 |  | 0.10 |
|  | A4F1G1S(6)1 |  | 0.15 |  | 0.30 |  | 0.10 |  | 0.10 |
|  | A2G(4,4)2S(6,6)2 |  | 0.45 |  | 1.10 |  | **2.00** |  | **2.30** |
|  | F(6)A2G1G(3Sul)1S(3,3)2 |  | 0.65 |  | 0.50 |  | 0.30 |  | 0.60 |
|  | A3BG(4,4)2S(3,6)2 |  | 0.05 |  | 0.15 |  | 0.10 |  | 0.05 |
|  | A2BG(4,4)2S(6,6)2 |  | 0.07 |  | 0.25 |  | 0.10 |  | 0.05 |
|  | F(6)A2BG(4,4)2S(3,6)2 |  | 0.22 |  | 0.25 |  | 0.10 |  | 0.10 |
|  | A4BG(4,4)2S(6)1 |  | 0.01 |  | 0.20 |  | 0.10 |  | 0.01 |
|  | F(6)A2F1G(3)2S(6)1 |  | 0.10 |  | 0.05 |  | 0.20 |  | 0.20 |
|  | A3G(3,3,3)3S(6)1 |  | 0.17 |  | 0.10 |  | 0.20 |  | 0.01 |
| **26** | F(6)A4G(3,3,3)3 | **9.91** | 0.25 | **9.91** | **0.30** | **9.98** | 0.20 | **9.94** | 0.20 |
|  | A3BG(4,4,4)3S(6)1 |  | 0.01 |  | 0.01 |  | 0.05 |  | 0.01 |
|  | F(6)A3BG(4,4,4)3S(3)1 |  | 0.01 |  | 0.01 |  | 0.05 |  | 0.01 |
|  | F(6)A4G(3,3)2S(6)1 |  | 0.01 |  | 0.01 |  | 0.10 |  | 0.08 |
|  | F(6)A3G(4,4,4)3S(3)1 |  | 0.01 |  | 0.01 |  | 0.18 |  | 0.11 |
|  | F(6)A4BG(4,4,4)3 |  | **0.60** |  | **0.30** |  | **0.50** |  | **0.60** |
| **27** | F(6)A2G1G(3Sul)1S(3,6)2 | **10.06** | 0.01 | **10.05** | 0.10 | **10.10** | 0.01 | **10.06** | 0.05 |
|  | A3BG(4,4,4)3S(3,3)2 |  | 0.01 |  | 0.01 |  | 0.01 |  | 0.01 |
|  | A3BG(4,4)2S(6,6)2 |  | 0.01 |  | 0.01 |  | 0.01 |  | 0.01 |
|  | A4BG(4,4,4)3S(3)1 |  | 0.01 |  | 0.01 |  | 0.01 |  | 0.02 |
|  | A4BF1G1S(6)1 |  | 0.01 |  | 0.05 |  | 0.01 |  | 0.02 |
|  | A4G3GlcNAc3 |  | **0.30** |  | **0.30** |  | **0.30** |  | **0.30** |
|  | F(6)A4BG(4,4)2S(3)1 |  | 0.01 |  | 0.05 |  | 0.01 |  | 0.01 |
|  | F(6)A3BG(4,4,4)3S(6)1 |  | 0.01 |  | 0.05 |  | 0.01 |  | 0.01 |
|  | A4BG(4,4)2S(3,3)2 |  | 0.05 |  | 0.03 |  | 0.05 |  | 0.05 |
|  | F(6)A3F1G(4)2GalNAc1S(3)1 |  | 0.01 |  | 0.02 |  | 0.20 |  | 0.20 |
| **28** | A4BG(4,4,4,4)4 | **10.13** | 0.01 | **10.14** | 0.05 | **10.19** | 0.01 | **10.16** | 0.40 |
|  | F(6)A3F1G(4)2GalNAc1S(3)1 |  | 0.10 |  | 0.20 |  | 0.60 |  | 0.79 |
|  | A3G(4)3Lac1 |  | 1.00 |  | 1.40 |  | 1.00 |  | 0.80 |
|  | F(6)A2BG2S(6,6)2 |  | 0.10 |  | 0.10 |  | 0.20 |  | **1.45** |
|  | F(6)A2G(4,4)2S(6,6)2 |  | 1.15 |  | 0.40 |  | **2.20** |  | 0.40 |
|  | F(6)A3F2G(4)3 |  | 0.24 |  | 0.20 |  | 0.20 |  | 0.40 |
|  | F(6)A3G(4,4,4)3S(6)1 |  | 0.07 |  | 0.10 |  | 0.20 |  | 0.30 |
|  | F(6)A4G(4,4,4,4)4 |  | **1.40** |  | **1.90** |  | 0.40 |  | 0.80 |
| **29** | F(6)A4G(4,4,4,4)4 | **10.29** | **0.45** | **10.30** | **0.60** | **10.35** | 0.30 | **10.31** | 0.10 |
|  | F(6)A3G(4,4,4)3S(6)1 |  | 0.01 |  | 0.01 |  | 0.10 |  | 0.01 |
|  | A3BG3S(3,6)2 |  | 0.01 |  | 0.01 |  | 0.20 |  | 0.01 |
|  | F(6)A3BG3S(3,3)2 |  | 0.01 |  | 0.01 |  | 0.01 |  | 0.01 |
|  | M9-Glc |  | 0.43 |  | 0.43 |  | **0.33** |  | **0.26** |
| **30** | F(6)A2G1G(3Sul)1S(6,6)2 | **10.44** | 0.01 | **10.44** | 0.01 | **10.49** | 0.05 | **10.46** | 0.05 |
|  | F(6)A3G(4)3Lac1 |  | **0.22** |  | **0.23** |  | **0.35** |  | **0.23** |
|  | F(6)A4BG(4,4)2S(6)1 |  | 0.01 |  | 0.01 |  | 0.04 |  | 0.01 |
|  | F(6)A4G(3,3,3)3S(3)1 |  | 0.05 |  | 0.01 |  | 0.06 |  | 0.03 |
|  | A4BG(4,4,4)3S(6)1 |  | 0.05 |  | 0.05 |  | 0.02 |  | 0.01 |
|  | A4BG(4,4,4)3S(3,3)2 |  | 0.05 |  | 0.01 |  | 0.02 |  | 0.01 |
|  | A4BG(4,4)2S(3,6)2 |  | 0.05 |  | 0.01 |  | 0.02 |  | 0.01 |
|  | F(6)A4F2G(4)3 |  | 0.10 |  | 0.05 |  | 0.09 |  | 0.10 |
|  | F(6)A4BG(4,4)2S(3,3)2 |  | 0.01 |  | 0.05 |  | 0.03 |  | 0.01 |
|  | A3BG(4,4,4)3S(6,6)2 |  | 0.01 |  | 0.05 |  | 0.03 |  | 0.01 |
|  | F(6)A3G(4)2GalNAc1S(3,3)2 |  | 0.10 |  | 0.10 |  | 0.05 |  | 0.20 |
|  | F(6)A3G(4,4,4)3S(3,3)2 |  | 0.10 |  | 0.15 |  | 0.02 |  | 0.05 |
| **31** | F(6)A3G(4,4,4)3S(3,3)2 | **10.68** | **0.52** | **10.70** | **0.80** | **10.74** | **0.50** | **10.71** | 0.35 |
|  | F(6)A4BG(4,4,4,4)4 |  | 0.30 |  | 0.17 |  | 0.19 |  | **0.36** |
|  | F(6)A4BG(4,4)2S(3,6)2 |  | 0.05 |  | 0.01 |  | 0.01 |  | 0.10 |
|  | A3G(3,3,3)3S(6,6)2 |  | 0.30 |  | 0.30 |  | 0.25 |  | 0.21 |
| **32** | F(6)A4BG(4,4,4)3S(3)1 | **10.76** | 0.20 | **10.78** | 0.05 | **10.83** | 0.20 | **10.80** | 0.14 |
|  | F(6)A3BG(4,4,4)3S(3,6)2 |  | 0.03 |  | 0.01 |  | 0.01 |  | 0.01 |
|  | A3BG(4,4,4)3S(3,3,3)3 |  | 0.03 |  | 0.05 |  | 0.01 |  | 0.01 |
|  | A4BG(4,4)2S(6,6)2 |  | 0.06 |  | 0.05 |  | 0.05 |  | 0.05 |
|  | F(6)A3G(4,4,4)3S(3,6)2 |  | **0.60** |  | **0.90** |  | **0.49** |  | **0.44** |
| **33** | F(6)A4G(3,4,4,4)4S(3)1 | **10.91** | **0.51** | **10.92** | 0.01 | **10.97** | 0.20 | **10.94** | 0.10 |
|  | F(6)A4BG(4,4)2S(6,6)2 |  | 0.29 |  | **0.30** |  | 0.09 |  | 0.16 |
|  | F(6)A3G2GalNAc1S(6,6)2 |  | 0.00 |  | 0.20 |  | 0.00 |  | 0.00 |
|  | F(6)A4BG(4,4,4)3S(6)1 |  | 0.30 |  | 0.30 |  | **0.39** |  | **0.40** |
|  | A3G(4)3Lac1S(3)1 |  | 0.00 |  | 0.10 |  | 0.00 |  | 0.00 |
| **34** | F(6)A4BG(4,4,4)3S(6)1 | **11.01** | 0.10 | **11.03** | **0.20** | **11.09** | 0.10 | **11.05** | 0.10 |
|  | F(6)A3BG(4,4,4)3S(3,3,3)3 |  | 0.08 |  | 0.05 |  | 0.05 |  | 0.01 |
|  | F(6)A3G(4,4,4)3S(6,6)2 |  | **0.15** |  | 0.05 |  | **0.15** |  | **0.16** |
| **35** | F(6)A3F2G(4,4,4)3S(3,3)2 | **11.24** | 0.03 | **11.24** | 0.20 | **11.29** | 0.20 | **11.25** | 0.10 |
|  | F(6)A3BG(4,4,4)3S(6,6)2 |  | 0.03 |  | 0.20 |  | 0.25 |  | 0.20 |
|  | A3BG(4,4,4)3S(3,3,6)3 |  | 0.10 |  | 0.20 |  | **0.30** |  | 0.20 |
|  | A4G(4,4,4)4Lac1 |  | **0.70** |  | **0.60** |  | 0.29 |  | 0.49 |
|  | A3G(4,4,4)3S(3,3,6)3 |  | 0.10 |  | 0.20 |  | 0.20 |  | **0.64** |
|  | F(6)A3G3S(3,3,3)3 |  | 0.00 |  | 0.10 |  | 0.00 |  | 0.00 |
|  | F(6)A4G(4,4,4,4)4S(6)1 |  | 0.20 |  | 0.01 |  | 0.30 |  | 0.10 |
| **36** | F(6)A4G(4,4,4,4)4S(6)1 | **11.47** | 0.01 | **11.46** | 0.02 | **11.51** | 0.15 | **11.47** | 0.09 |
|  | F(6)A3BG3S(3,3,6)3 |  | 0.03 |  | 0.01 |  | 0.04 |  | 0.01 |
|  | A3G(4)3Lac2 |  | **0.75** |  | **0.80** |  | **0.35** |  | **0.21** |
|  | F(6)A4G(4,4,4)4Lac1 |  | 0.32 |  | 0.40 |  | 0.20 |  | 0.20 |
|  | F(6)A4G(4,4,4,4)4S(3,3)2 |  | 0.02 |  | 0.01 |  | 0.15 |  | 0.09 |
|  | F(6)A4F2G(4,4)4 |  | 0.12 |  | 0.10 |  | 0.01 |  | 0.10 |
| **37** | A3G(4,4,4)3S(3,6,6)3 | **11.62** | 0.05 | **11.61** | 0.20 | **11.64** | 0.05 | **11.61** | 0.05 |
|  | F(6)A3F2G(4,4,4)3S(3,6)2 |  | 0.02 |  | 0.01 |  | 0.20 |  | 0.05 |
|  | F(6)A3G(4)3Lac2 |  | 0.25 |  | **0.40** |  | 0.22 |  | **0.30** |
|  | A3BG3S(3,6,6)3 |  | 0.10 |  | 0.10 |  | 0.01 |  | 0.05 |
|  | F(6)A3G(4,4,4)3S(3,3,6)3 |  | **0.52** |  | 0.05 |  | **0.39** |  | 0.28 |
| **38** | F(6)A4F2G(4,4)4S(3)1 | **11.95** | 0.07 | **11.92** | 0.10 | **11.97** | 0.05 | **11.93** | 0.17 |
|  | F(6)A3BG(4,4,4)3S(3,6,6)3 |  | 0.10 |  | 0.10 |  | 0.01 |  | 0.21 |
|  | F(6)A4G(4,4)4Lac2 |  | **0.39** |  | 0.15 |  | 0.05 |  | 0.03 |
|  | F(6)A4G(4,4,4,4)4S(3,6)2 |  | 0.25 |  | 0.10 |  | 0.16 |  | **0.25** |
|  | F(6)A4F2G(4,4,4)4Lac1 |  | 0.15 |  | 0.15 |  | 0.05 |  | 0.04 |
|  | F(6)A3G(4,4,4)3S(3,6,6)3 |  | 0.32 |  | **0.80** |  | **0.80** |  | 0.20 |
| **39** | A3G(4,4,4)3S(6,6,6)3 | **12.16** | 0.20 | **12.15** | **0.40** | **12.22** | 0.05 | **12.12** | 0.01 |
|  | A3BG(4,4,4)3S(6,6,6)3 |  | 0.10 |  | 0.20 |  | 0.01 |  | 0.10 |
|  | F(6)A4F2G(4,4)4S(6)1 |  | 0.05 |  | 0.20 |  | 0.05 |  | 0.05 |
|  | F(6)A4G(4,4,4,4)4S(6,6)2 |  | 0.10 |  | 0.05 |  | 0.05 |  | 0.05 |
|  | F(6)A4G(4,4,4,4)4S(3,3,3)3 |  | 0.10 |  | 0.05 |  | 0.05 |  | 0.10 |
|  | F(6)A3G(4,4,4)3S(6,6,6)3 |  | **0.30** |  | 0.20 |  | **0.30** |  | 0.20 |
|  | F(6)A3BG(4,4,4)3S(6,6,6)3 |  | 0.30 |  | 0.10 |  | 0.30 |  | 0.20 |
|  | F(6)A4G(4,4,4)4Lac1S(3)1 |  | 0.10 |  | 0.10 |  | 0.10 |  | **0.34** |
| **40** | F(6)A4G(4,4,4,4)4S(3,3,6)3 | **12.64** | 0.27 | **12.59** | **0.80** | **12.62** | 0.10 | **12.61** | 0.30 |
|  | A3G3Lac3 |  | **0.70** |  | 0.40 |  | **0.23** |  | **0.40** |
|  | F(6)A4G(4,4,4)4Lac1S(6)1 |  | 0.15 |  | 0.01 |  | 0.05 |  | 0.20 |
|  | F(6)A4F2G(4,4)4S(6,6)2 |  | 0.00 |  | 0.00 |  | 0.10 |  | 0.00 |
| **41** | F(6)A4G(4,4,4,4)4S(3,6,6)3 | **12.81** | 0.14 | **12.73** | **0.20** | **12.90** | **0.10** | **12.88** | 0.07 |
|  | F(6)A3G(4)3Lac3 |  | **0.25** |  | **0.20** |  | 0.09 |  | **0.08** |
|  | F(6)A4G(4,4,4,4)4S(3,3,3,3)4 |  | 0.17 |  | 0.10 |  | 0.05 |  | 0.07 |
|  | F(6)A4G(4,4)3Lac1S(6,6)2 |  | 0.00 |  | 0.00 |  | 0.00 |  | **0.08** |
| **42** | F(6)A4G(4,4,4)4Lac1S(3,3)2 | **13.11** | **0.24** | **13.09** | **0.53** | **13.14** | **0.21** | **13.14** | **0.15** |
| **43** | F(6)A4G(4,4,4)4Lac1S(3,6)2 | **13.33** | 0.01 | **13.23** | **0.39** | **13.22** | 0.07 | **13.23** | 0.07 |
|  | F(6)A4F2G(4,4)4Lac2 |  | **0.35** |  | 0.30 |  | **0.13** |  | **0.14** |
|  | F(6)A4G4Lac2S(6)1 |  | 0.04 |  | 0.30 |  | 0.07 |  | 0.07 |
| **44** | F(6)A4G4Lac1S(6,6)2 | **13.71** | 0.01 | **13.61** | 0.02 | **13.64** | 0.04 | **13.64** | 0.02 |
|  | F(6)A4F2G4Lac1S(3,3)2 |  | 0.01 |  | 0.01 |  | 0.04 |  | 0.02 |
|  | F(6)A4G(4,4,4,4)4S(3,3,6,6)4 |  | 0.10 |  | 0.01 |  | 0.04 |  | 0.02 |
|  | A4G(4)4Lac3 |  | **0.50** |  | **0.60** |  | **0.10** |  | **0.16** |
|  | F(6)A4G(4,4,4,4)4S(6,6,6)3 |  | 0.02 |  | 0.01 |  | 0.04 |  | 0.02 |
|  | F(6)A4G(4,4)4Lac2S(6)1 |  | 0.00 |  | 0.00 |  | 0.01 |  | 0.00 |
| **45** | F(6)A4G(4)4Lac3 | **13.90** | 0.08 | **13.72** | 0.07 | **13.86** | **0.09** | **13.82** | **0.06** |
|  | F(6)A4G(4,4,4)4Lac1S(3,3,3)3 |  | **0.10** |  | **0.20** |  | 0.01 |  | 0.02 |
|  | F(6)A4F2G(4,4,4)4Lac1S(3,6)2 |  | 0.03 |  | 0.02 |  | 0.02 |  | 0.04 |
|  | F(6)A4F2G(4,4)4Lac2S(3)1 |  | 0.00 |  | 0.10 |  | 0.00 |  | 0.00 |
|  | F(6)A4F2G4Lac1S(3,3,3)3 |  | 0.03 |  | 0.05 |  | 0.02 |  | 0.04 |
| **46** | F(6)A4F2G(4,4)4Lac2S(6)1 | **14.41** | 0.02 | **14.24** | 0.10 | **14.30** | 0.01 | **14.36** | 0.01 |
|  | F(6)A4G4Lac2S(6,6)2 |  | 0.00 |  | 0.05 |  | 0.00 |  | 0.00 |
|  | A4G4Lac4 |  | **0.28** |  | **0.20** |  | **0.08** |  | **0.11** |
|  | F(6)A4F2G4Lac1S(3,6)2 |  | 0.00 |  | 0.00 |  | 0.01 |  | 0.00 |
|  | F(6)A4G(4,4)4Lac2S(3,3,3)3 |  | 0.02 |  | 0.02 |  | 0.01 |  | 0.01 |
|  | F(6)A4F2G(4,4,4)4Lac1S(6,6)2 |  | 0.01 |  | 0.18 |  | 0.01 |  | 0.01 |
|  | F(6)A4F2G(4,4,4)4Lac1S(3,3,6)3 |  | 0.05 |  | 0.10 |  | 0.01 |  | 0.01 |
| **47** | F(6)A4G(4,4)4Lac2S(3,3,6)3 | **14.94** | 0.02 | **14.70** | 0.10 | **14.75** | 0.04 | **14.84** | 0.01 |
|  | F(6)A4G4Lac4 |  | **0.26** |  | **0.36** |  | **0.08** |  | **0.08** |
